# Untargeted metabolomics of mature sorghum [*Sorghum bicolor* (L.) Moench] grain reveals metabolites associated with antimicrobial activity against *Clostridium perfringens*

**DOI:** 10.3389/fpls.2026.1821654

**Published:** 2026-05-11

**Authors:** María Antonella Conti, Carolina Ballén-Taborda, Corey D. Broeckling, Richard Elmer Boyles

**Affiliations:** 1 Pee Dee Research and Education Center, Clemson University, Florence, SC, United States; 2 Bioanalysis and Omics Center, Analytical Resources Core, Colorado State University, Fort Collins, CO, United States

**Keywords:** antimicrobial metabolites, *Fusarium*, non-tannin sorghum, qPCR, QTL mapping, untargeted metabolomics

## Abstract

The poultry industry is facing challenges with antibiotic-resistant bacteria, particularly *Clostridium perfringens*, the causal agent of necrotic enteritis. Sorghum grain, well-known for its high phenolic content that can provide consumers with health benefits, offers a great opportunity for poultry feed. While condensed tannins in sorghum grain have an antinutritional effect by binding proteins and decreasing nutrient absorption in the poultry gut, their concentration is genetically controlled, and tannin-free sorghums exist. This study analyzed a non-tannin sorghum recombinant inbred line (RIL) population consisting of 189 individuals along with the two parents using LC-MS untargeted metabolomics to investigate its antimicrobial (AM) activity against *C. perfringens* measured by 1) a minimum inhibitory concentration (MIC) analysis with phenol extract of the grain, and 2) a qPCR-based approach to measure bacterium inhibition with *in vitro* enzymatically digested grain. Results showed that RILs differing in AM activity exhibited distinct metabolic profiles, which varied across methods. The qPCR assay classification revealed a greater number of pathways enriched, with alpha-linolenic acid metabolism being the most significant. Meanwhile, flavonoid biosynthesis was the most enriched pathway for the MIC-based AM trait. Metabolite selection analysis revealed that 13 metabolites were most relevant in separating high and low AM RILs, including two metabolites (C0153b and C0560) tentatively identified as *Fusarium* products, a naturally occurring pathogen in the tested environment. Only two of the 13 metabolites exhibited higher abundance in the high AM lines, C0540, a hydroxycinnamic acid, and C0916, a glycerophosphate. QTL mapping revealed that five metabolites (C0153b, C0560, C0540, C0916, and C0024) identified a major-effect QTL on chromosome 1 at 25.6 Mb, co-localizing with a previously reported QTL for the qPCR-based AM trait. Relative abundance for each of these five metabolites also mapped to a secondary, and in some cases, a tertiary QTL, indicating oligogenic control of metabolite biosynthesis that is associated with inhibition of *C. perfringens*. This work provides insight into the metabolic and genetic basis of AM activity in sorghum that can be leveraged to develop sorghum-based feed additives to improve poultry health using natural compounds.

## 1 Introduction

Sorghum [*Sorghum bicolor* (L.) Moench] grain is rich in bioactive compounds (Bravo, 1998) with antioxidant, anti-inflammatory, and antimicrobial properties (Rao et al., 2018), making it a valuable and functional food providing health benefits to humans and animals (Ridaura et al., 2013; Singh et al., 2017). Phenolic compounds, particularly abundant in sorghum compared to other cereal crops, contribute to its bioactive properties (Awika, 2011; Kang et al., 2016). The content and profile of these compounds vary by the genotype, pericarp color, testa pigmentation, and environmental conditions during crop growth (Wu et al., 2017). The structural localization of phenolic compounds in the grain affects their bioavailability, metabolism, and absorption, with bound phenolic compounds having low bioaccessibility. Bound compounds can become bioaccessible during the digestion process throughout the gastrointestinal tract due to changes in pH, enzyme activity, and by being metabolized through microbial intervention in the gut (de Morais Cardoso et al., 2017; Domínguez-Avila et al., 2017; Grgić et al., 2020).

Phenolic compounds can be categorized into flavonoids and nonflavonoid compounds. The main group of nonflavonoids is phenolic acids, while some of the most important subclasses of flavonoids are flavones, flavanones, anthocyanins and proanthocyanidins (*i.e.*, tannins). These compounds are synthesized via the shikimate, phenylpropanoid, and flavonoid pathways, which are downstream of one another (Crozier et al., 2006; Jaganath and Crozier, 2010), and many exhibit beneficial properties (Hartzfeld et al., 2002; Makanjuola et al., 2018). Among flavonoids, condensed tannins are particularly noteworthy because they play a key role in plant defense against pathogens and predators (mainly birds) (Kaufman et al., 2013), and possess high antioxidant potential (Waniska et al., 1989; Serna-Saldivar, 2016). However, they also have antinutritive effects, reducing protein digestibility and digestive enzyme activity. In sorghum, tannins are found in genotypes with a pigmented testa, and their presence and quantity are genetically controlled (Hahn and Rooney, 1986; Awika and Rooney, 2004) by two loci, *B1* and *B2* (Dykes et al., 2005), whose underlying genes have been identified as *Tannin1* (*Tan1*) and *Tannin2* (*Tan2*), respectively (Wu et al., 2012, Wu et al., 2019). Grain lacking condensed tannins has been shown to provide health benefits to animals while avoiding the drawbacks associated with these compounds (Garcia et al., 2013; Moritz et al., 2023; Sasia et al., 2023).

The poultry industry, particularly concerned with antibiotic resistance, could benefit from incorporating non-tannin sorghum as a feed ingredient, as it can help maintain birds’ gut health. *Clostridium perfringens*, a Gram-positive, anaerobic bacterium part of the normal gut microbiota (Vierheilig et al., 2013), can produce toxins (Songer, 1996) and cause necrotic enteritis, one of the most widespread and problematic diseases in the industry. The presence of an antibiotic-resistant strain of *C. perfringens* in a poultry farm could potentially be transferred from animals to humans (Diaz Carrasco et al., 2016). Non-tannin sorghums with various grain color pigmentation have been shown to significantly reduce the concentration of *C. perfringens* in the microbiome of poultry without negatively impacting bird performance, with flavonoids and phenolic acids as the main bioactive compounds involved (Garcia et al., 2013; Moritz et al., 2023; Sasia et al., 2023).

Sorghum produces defense-related metabolites in response to pathogen attacks (Boddu et al., 2004; Poloni and Schirawski, 2014; Piasecka et al., 2015; Nida et al., 2019), known as phytoalexins and regulated by plant hormones (Meyer et al., 2016). A key class of phytoalexins in sorghum are 3-deoxyanthocyanidins (3-DAs) (Boddu et al., 2004), with apigeninidin and luteolinidin being the most abundant. The accumulation of 3-DAs is triggered by the phytohormone methyl jasmonate (Liu et al., 2010), and the grain pigmentation gene *YELLOWSEED1* (*Y1*) (*Sobic.001G398100*) is involved in 3-DAs synthesis, which has been reported to be involved in resistance to grain mold (Nida et al., 2019) and *Colletotrichum sublineolum*, the causal agent of anthracnose in sorghum (Boddu et al., 2005). In addition to inducible phytoalexins, sorghum also contains constitutive metabolites, or phytoanticipins, which persist in mature grain after harvest (Osbourn, 1996; Piasecka et al., 2015; Meyer et al., 2016). These compounds represent the metabolite pool that most likely influences poultry health, and the antimicrobial (AM) activity against *C. perfringens* observed in poultry fed with sorghum grain (Moritz et al., 2023) is plausibly attributed to these constitutive metabolites. Many constitutive bioactive compounds are associated with pericarp color, with pigmented grain having higher levels of flavonoids (Dykes et al., 2009, 2011; Yang et al., 2015; Awika, 2017).

In this study, the metabolic profile of mature sorghum grain from a non-tannin recombinant inbred line (RIL) population was evaluated using untargeted metabolomics to identify antimicrobial compounds effective against *C. perfringens*. For the metabolites that showed association with AM activity, a QTL analysis was conducted to identify the underlying genetic regions. This work provides valuable insight into the genetic and metabolic basis of sorghum grain AM activity. It lays the foundation for developing genetic markers or biomarkers to guide breeding programs aimed at increasing bioactive compound concentrations in grain, offering potential for developing sustainable solutions to combat AM resistance in poultry.

## 2 Materials and methods

### 2.1 Plant material, experimental design, and field trial management

An established RIL population of 189 RILs from the cross between Tx2911 and P850029 was provided by Dr. William Rooney of the Texas A&M University sorghum breeding program. The RIL population is considered tannin-free since both parents lack a pigmented testa, meaning they have no condensed tannins. The parental lines possess some contrasting characteristics. Tx2911 has a red pericarp, a high phenolic content, harbors resistance to sorghum grain mold (SGM) and grain weathering, and carries the intensifier gene (Esele et al., 1995), which enhances the brightness of the red pericarp. Conversely, P850029 has a white pericarp color, is a high-lysine line, and possesses a highly digestible protein trait (Weaver et al., 1998), which has been shown to increase grain mold susceptibility (Gould et al., 2009). These contrasting traits provide a useful framework for examining the genetic and metabolic basis of AM activity in the grain, as they create variation in metabolites that may contribute to AM effects.

The F_6_ RILs and the two parents (191 total genotypes) were planted in 2023 at the Clemson University Pee Dee Research and Education Center in Florence, South Carolina, USA. The experiment was planted using an alpha-lattice design with two field replicates. Field trial and management are fully detailed in Conti et al. (2026).

### 2.2 Harvest and post-harvest

At physiological maturity, a total of ten primary panicles were collected by hand from the center of each plot to obtain a pure seed source for subsequent analysis. Pericarp color was visually assessed at a plot level and classified as W (white), R (red), bR (bright red), or their combination (*i.e.*, RW, bR-W, or bR-R) when clear segregation was observed within the plot. For plots exhibiting RW or bR-W pericarp colors, panicles corresponding to each color class were collected separately (five panicles per color), processed and analyzed as independent samples.

The ten panicles from each plot were collectively threshed using an Almaco BT14 belt thresher and cleaned grain was bulked into a single envelope. A 50 g grain sample from each plot was ground to 1 mm particle size with a CT 193 Cyclotec Sample Mill (FOSS North America). To assess AM activity against *C. perfringens*, sub-samples of 500 mg and 5 g were used for a minimum inhibitory concentration (MIC) and an *in vitro* digestion with qPCR analysis, respectively. An additional 2 g subsamples were sent to the Colorado State University Analytical Resource Core (ARC) facility for untargeted metabolomics.

### 2.3 AM activity phenotyping

#### 2.3.1 Minimum inhibitory concentration analysis

The MIC analysis is fully detailed in Conti et al. (2026). In brief, phenolic compounds were extracted from each ground grain sample of 500 mg using a total of 10 ml of 70% acetone. The supernatants were then dried completely under nitrogen evaporation in a heating block at 30°C. The dried extracts were weighed and resuspended in 40% DMSO. The extract concentration of each sample varied due to differences in the extraction efficiency and grain heterogeneity, with concentrations ranging from 14.9 mg ml^-1^ – 32.9 mg ml^-1^ (after removing statistical outliers). Moreover, taking the exact weight of all the extract samples and resuspending them in an equal volume of DMSO would have sufficed; however, the limited amount of sample extract was a major limitation. To account for the variation in the concentrations during MIC analysis, the extracts were serially diluted in double-fold increments, which normalized the inherent variation in sample concentrations to ensure the MIC reflected the true antimicrobial activity of each extract, avoiding bias from starting concentration differences. Dilutions were made using Brucella blood broth, and 10 µl of 10^6^ CFU ml^-1^ of *Clostridium perfringens* (strain CP6) was added to each well to obtain a final bacterial density of 10^5^ CFU ml^-1^ in each well. This was done in triplicate. After incubation under anaerobic conditions at 37°C for 24 hours, the MIC was recorded as the lowest extract concentration that inhibited the growth of CP6, as evidenced by the presence of turbidity and cell pellets in the wells. The results were validated by plating out samples from each well on brain heart infusion agar and incubating anaerobically for 24 hours at 37°C to determine isolate viability. The lowest concentration of the samples with no colonies or fewer than 30 CFU ml^-1^ was considered the MIC value.

#### 2.3.2 *In vitro* enzymatic digestion and qPCR

The *in vitro* enzymatic digestion and qPCR are described in Conti et al. (2026). Briefly, each ground grain sample was digested *in vitro* using 0.2 ml *Aspergillus niger* amyloglucosidase (E-AMGDF, 3, 260 U/ml, Megazyme). Following digestion, the material dialyzed against distilled water for 72 hours at 4 °C with a water change every 12 h, and the dialyzed samples were then lyophilized. The digested and lyophilized seed components were resuspended in 30 ml of ddH20 and 575 µl of the resuspended sample was added to a 1 ml-deep well (96-well plate) with 65 µl Brain Heart Infusion broth (BHI). The plates were then placed into the anaerobic chamber and reduced at 4 °C with an anaerobic gas generator (Mitsubishi™ AnaeroPack-Anaero, Japan) for 3 days. After reduction, each well was inoculated with 100 μL of a master culture of *C. perfringens* CP6. After inoculation, the *in vitro* fermentations were incubated anaerobically at 37 °C for 8 h. The terminal bacterial growth at the end of fermentation was monitored by qPCR. DNA extractions were performed on pellets from the fermentation using the BioSprint 96 One-For-All kit (384) (Qiagen). DNA was also extracted from dilutions of a *C. perfingens* CP6 culture for use as a standard curve. qPCR reactions were performed using the plc gene from *C. perfringens* with previously validated forward (Cper-plc508-F CCGTTGATAGCGCAGGACA) and reverse primers (Cper-plc508-R CCCAACTATGACTCATGCTAGCA) using PCR conditions specified (Nagpal et al., 2015). Quantitative PCR reactions were done in duplicate for each fermentation reaction.

For each biological sample (sorghum genotype), three technical replicates were taken for fermentation. From each fermentation replicate, two qPCR measurements were conducted, giving a threshold cycle (C_t_) value that was then log_10_ transformed based on the normalization curve to obtain log_10_(CFU) values. The mean of the two qPCR results was calculated. There were 20 qPCR outliers detected based on normal quantile plot and box plot of conditional residuals in JMP (JMP Statistical Discovery LLC, 2025) that were removed for subsequent analyses.

### 2.4 LC-MS untargeted metabolomics

#### 2.4.1 Sample preparation

Twenty mg of ground grain was weighed, and 2 mL of 80% MeOH was added. The samples were sonicated at room temperature for 1 min, vortexed for 10 min, then centrifuged at 1, 900 x g for 15 min. From this 2 mL extract, 1 mL was aliquoted and dried down under nitrogen for 1 h at 40°C and stored at -80°C until the day of run. An additional 200 µL were aliquoted from each extract and pooled to generate QC samples. Each day before running samples on the LC/MS, 100 µL of 80% MeOH was added to each vial, vortexed at 4°C for 30 min, then added to a HPLC vial insert. Finally, the samples in vials were centrifuged at 3000 x g for 15 min before placement on the autosampler.

#### 2.4.2 LC-MS analysis

A 1 µL aliquot was injected onto a Waters Acquity UPLC system. Separation was achieved using a Waters ACQUITY UPLC Premier T3 1.7μm Column (1.7 μM, 2.1 x 100 mm) coupled to a Xevo G2-XS Q-TOF, using a gradient from solvent A (Water + 0.1% formic acid) to solvent B (Acetonitrile with 0.1% Formic Acid) and a flow rate of 0.6 mL min^-1^. The column and samples were held at 45 °C and 6 °C, respectively. Data was acquired in positive polarity, with data independent MS^E^ MS/MS mode, in sensitivity mode using extended dynamic range. Positive ionization mode was applied as it was observed to detect more metabolites than negative ionization mode, and we wished to detect as many metabolite classes as possible. The mass range was 50–1200 *m/z*, with 0.1 s scan time alternating between MS1, with 6 V collision energy and MS^E^ with collision energy ramped 15–30 V. Calibration was performed using sodium formate with 1 ppm mass accuracy. The capillary voltage was held at 700 V in positive mode. The source temperature was held at 150 °C and the nitrogen desolvation temperature at 450 °C with a desolvation flow rate of 1000 L h^-1^. A lockspray reference mass (LeuEnk, *m/z* 556.2771) was used for mass drift correction, with a scan interval of 40 s, an acquisition time of 0.1 s per scan, and signal averaging over three scans. MS^E^ (DIA) MS/MS injections were supplemented by 20 injections of 4 samples, using data-dependent MS/MS acquisition mode implementing iterative exclusion to increase depth of coverage. Experimental sample run order was completely randomized. A pooled quality control sample was injected approximately every 7^th^ injection.

#### 2.4.3 Data processing

The software XCMS (Smith et al., 2006, Tautenhahn et al., 2008) version 3.22.0 was used to process raw data using R v4.3.1. RAMClustR version 1.3.0 in R version 4.3.1 (2023-06-16) was used to normalize, filter, and group features into spectra. XCMS (Smith et al., 2006; Tautenhahn et al., 2008) output data was transferred to a ramclustR object using the *rc.get.xcms.data* function. Feature data was extracted using the *xcms featureValues* function. Features with missing values were replaced with small values simulating noise. For each feature, the minimum detected value was multiplied by 0.5. Noise was then added using a factor of 0.5, and its absolute value was used to ensure that only non-negative values carried forward. Variance in quality control samples was described using the *rc.qc* function within *ramclustR*. Features were normalized by linearly regressing run order versus QC feature intensities to account for instrument signal intensity drift. Only features with a regression *p*-value< 0.05 and an *r*^2^ > 0.1 were corrected. Of 44, 427 features, 26, 531 were corrected for run order effects. Features that failed to demonstrate a signal intensity of at least 2-fold greater in QC samples than in blanks were removed from the feature dataset. 17, 480 of 44, 427 features were removed. Features were filtered based on their QC sample coefficient of variation (CV) values. Only features with a CV value less than or equal to 1 in MSdata set were retained. 3, 466 of 26, 947 features were removed. Features were clustered using the *ramclustR* algorithm (Broeckling et al., 2014). Molecular weight was inferred from in-source spectra (Broeckling, 2026) using the *do.findmain* function, which calls the *interpretMSSpectrum* package (Jaeger et al., 2016). Specific parameters used for XCMS, *ramclustR* algorithm, and *do.findmain* function are listed in the **Supplementary Material**.

#### 2.4.4 Annotation

Annotation was manually performed for the most important metabolites, tentatively assigning structures to signals based on accurate mass, precursor isotope pattern, and taxonomy-informed structure scoring. While MS/MS data was available for some of the target analytes, these proposed structures did not have library spectral available for spectral matching. A taxonomy-informed subset of PubChem was generated using the *pubchem.bio* R package (Broeckling, 2026). The taxa chosen included the genera *Sorghum* and *Fusarium*, taxid = 4557 and 5506, respectively (Broeckling, 2026). The inclusion of *Fusarium* in the search space is based on knowledge that *Fusarium* is a common pathogen under field conditions. The resulting *pubchem.bio* taxonomy scores were used to prioritize the most likely structures based on accurate mass and similarity between the measured and predicted isotope patterns. When taxonomy scores were unavailable, compounds were manually interpreted based on accurate mass, isotope pattern, and literature evidence. The confidence level for the annotations was 2b.

### 2.5 Data transformation

The processed metabolic data was received in CSV format containing relative signal intensity values for 3, 780 compounds and 398 samples. The intensity peaks of raw metabolites were log_2_-transformed and scaled to a zero mean and unit variance (*Z*-score standardization). Absolute values exceeding four standard deviations (*|Z|* > 4) were winsorized to reduce the influence of extreme outliers (Kwak and Kim, 2017). A total of 1, 481 data points (from 1, 036 metabolites and 147 genotypes) were above the threshold. The raw peaks of outliers were replaced by the nearest non-outlier intensity peak. After winsorization, there were still 77 datapoints with *|Z|* > 4, but they were left as is since they represented ~ 0.01% of total data points. The adjusted raw intensity peaks were then log_2_-transformed, and the mean was calculated for biological replicates and auto-scaled.

### 2.6 Multivariate modeling, univariate analysis and statistical analysis

#### 2.6.1 Multivariate analysis

Principal component analysis (PCA), partial least squares (PLS) regression and partial least squares discriminant analysis (PLS-DA) were performed for understanding metabolite variation across the RIL population and for identifying significant compounds associated with AM activity determined by two distinct detection methods: a MIC analysis and qPCR assay of sorghum grain pre-digested *in vitro* (for further details refer to Conti et al., 2026). Functions *pca, pls, and plsda* from the *mixOmics* (v6.31.4) package in R (Rohart et al., 2017) were used for calculating PCA, PLS, and PLS-DA, respectively. PLS-DA was performed for AM activity groups using the whole population and with only the extreme categories of the AM activity classification (*i.e.*, High AM and Low AM) for each detection method (Conti et al., 2026). The subsets of the extreme groups consisted of a total of 65 RILs for the MIC (17 Low AM and 48 High AM), and 114 RILs for the qPCR (41 Low AM and 73 High AM). PLS-DA was also performed for pericarp color, comparing pigmented grain (R, bR and bR-R) and white grain.

To evaluate models performance, M-fold cross-validation was performed using the *perf* function from the *mixOmics* package. The data was randomly partitioned into 10 folds, with models trained on nine folds and tested on the remaining one. This was repeated 50 times to ensure robust estimations of predictive performance. PLS-DA models’ performance were evaluated using error rate and area under the curve (AUC), with a higher AUC value (>0.5) indicating better discrimination between groups (Marrocco et al., 2008; Ghosh et al., 2020). PLS models were assessed using the cross-validated correlation coefficient (Q^2^) and the root-mean-squared error of prediction (RMSEP), where higher Q^2^ (>0.5) and a lower RMSEP indicate a better predictive ability (Kim et al., 2020; Xu et al., 2025). The significance of PLS-DA models was also assessed by comparing the AUC and error rate values with those of the null reference distribution obtained via 1, 000 permutation tests (Szymańska et al., 2011).

Because PC1 explained a greater percentage of total variation, variable importance in projection (VIP) scores were extracted from PC1 of PLS-DA and PLS models (Kim and Lee, 2026) using the *vip* function from the same package, and VIP ≥ 1 were used to determine the metabolites that most influenced the separation between groups, and best predicted the AM activity. Further, for each metabolite and each PLS-DA and PLS model, Student’s *t*-tests were performed between groups using the *t.test* function from the R package *stats*, and *p*-values were adjusted for false discovery rate (FDR) using the *p.adjust* function from the same package. However, raw *p*-values were used for the MIC detection method, as no metabolites were significant when applying the FDR.

#### 2.6.2 Univariate analysis

To identify differentially expressed metabolites (DEMs) between groups of RILs, fold changes (FC) were calculated between categories. For each metabolite, using the mean intensity peaks of biological replicates from the adjusted, untransformed data, the ratio between the mean of each group (*i.e.*, High AM and Low AM, and Pigmented and White pericarp) was calculated and log_2_-transformed. Subsequently, *t*-tests and *p*-values adjusted for FDR were calculated. A threshold value of two FC or one log_2_(FC) was used to determine up- and down-regulated metabolites between groups.

To observe the abundance level of the most influential compounds based on VIP scores and absolute log_2_(FC) values for the AM activity in each method, heatmaps were performed on the extreme RILs (High AM and Low AM) using the *pheatmap* function from the *pheatmap* package in R.

Repeatability was calculated as the Pearson pairwise correlation between biological replicates of the adjusted, untransformed data using the *cor.test* function from the *stats* package. The CV for each metabolite was calculated using the mean of the biological replicates of the adjusted untransformed intensities.

### 2.7 Variable selection

To identify metabolites that could serve as biomarkers for AM activity, variable selection was performed using sparse PLS-DA (sPLS-DA) and sparse PLS (sPLS). The *splsda* and *spls* functions from the *mixOmics* package in R were used for setting the models and *tune.splsda* function from the same package was then used to determine the optimal number of variables. Cross-validation was then performed using the *perf* function with validation model “Mfold”, 10 folds and 50 repeats. Variables were selected for QTL mapping based on stability values > 0.70 across cross-validation folds.

### 2.8 Pathway enrichment

Metabolic pathway enrichment was performed using the Functional Analysis module in MetaboAnalyst (6.0). For each comparison, a univariate *t*-test was first performed in R to identify compounds associated with a particular group, comparing each category to the rest of the population (*e.g.*, High AM against all other RILs– Low AM + Medium AM). A table containing *m/z*, retention time (rt), *p*-values (FDR-adjusted), and *t*-scores was then formatted according to MetaboAnalyst requirements for each comparison and uploaded for pathway analysis. This resulted in pathway enrichment for each group relative to the rest of the population, showing how each extreme stands apart from the population.

MetaboAnalyst Functional Analysis workflow utilizes the *mummichog* algorithm, enabling the prediction of metabolic pathways by matching *m/z* and retention time information with a reference metabolome. The metabolic reference model used was *Zea mays* from the KEGG global metabolic network, as it was the closest available metabolomic reference library at the time of analysis. The analysis was performed using the following parameters: positive ion mode, a tolerance of 10 ppm, and a *p*-value cutoff of 0.05. For presenting the results, relative pathway enrichment was scored and evaluated using the log_10_ of Expression Analysis System Explorer (EASE) instead of Fisher’s Exact Test (FET), which highlights pathway enrichment with a higher number of significant hits per empirical compounds (Hosack et al., 2003). A higher EASE score indicates enrichment of a specific group pathway relative to the other groups.

### 2.9 Genotyping

Leaf tissue of 187 RILs was sampled from roughly 2-week-old F_7_ seedlings grown from seed obtained from self-pollinated plots harvested in 2023. For RILs segregating for pericarp color, the color that was genotyped was unknown. Tissue was placed into 96-well plates and desiccated using a bed of silica beads by covering plates with a modified cheesecloth mat, inverting them, and placing them on top of the silica beads in sealed plastic containers. Following desiccation, plates were sent to Intertek ScanBi Diagnostics (Alnarp, Sweden) for DNA extraction and genotyping. Genome-wide single-nucleotide polymorphisms (SNPs) were generated using Diversity Arrays Technology (DArT), with reads aligned to the *Sorghum bicolor* v3.1 reference genome (McCormick et al., 2018). The received.csv file report with SNP information was first filtered and formatted in R and subsequently filtered and imputed in TASSEL. Parental lines Tx2911 and P850029 were each represented by five and four sample replicates, respectively. Markers with missing genotype calls in more than two parental replicates or exhibiting heterozygous calls were excluded (2, 644 markers removed). Additionally, 20 SNPs with inconsistent calls across parental samples were removed. The remaining 10, 656 SNPs were subject to imputation and filtering using the graphical interface of TASSEL 5 software (v5.2.96) (Bradbury et al., 2007). Missing data was imputed using FSFHap, which is optimized for biparental populations. Imputation was performed separately for each chromosome. SNP filtering was performed with the Window LD algorithm with thresholds of 0.05 for minimum minor allele frequency (MAF), 0.10 for maximum proportion of heterozygotes, 0.80 for maximum missing sites, while all other parameters were left at default settings. Following filtering, 2, 486 SNPs distributed across all chromosomes were retained for a final set of 186 RILs. Genotype calls were converted to ABH format and exported in CSV format for genomic analysis and mapping in R. Individual SNPs were designated by chromosome number and physical position in base pairs (bp), separated by an underscore (*e.g.*, 01_1234567).

### 2.10 QTL mapping

The genotype file with 2, 486 SNPs was read into R using the *read.cross* function from the package *qtl* (Broman and Sen, 2009), using “riself” as the cross type. Four individuals with more than 20% missing data were removed. For markers, segregation distortion was evaluated, and markers with *p*-value< 1e^-20^ were removed. Additionally, recombination fractions were calculated using the *est.rf* function and marker grouping was evaluated per chromosome using the *formLinkageGroups* function (max.rf = 0.25, min.lod = 6); single-marker groups were removed prior to map construction. A total of 182 RILs and 2, 448 markers were obtained. The genetic map was estimated with the function *est.map* with an error rate of 1e^-4^, map function “kosambi”, and tolerance of 1e^-4^. The total length of the genetic map was 1192.7 cM, with an overall average spacing of 0.5 cM.

The functions *calc.genoprob*, with map function “kosambi”, and *makeqtl* and *fitqtl* with the Haley-Knott regression were used for fitting models and getting the estimated and percent variances explained (PVE) for metabolites of interest following the model described in Equation 1:

(1) $$\text{y}=\sum_{i=1}^{t}X_{i}{\text{β}}_{i}+\text{ϵ}$$

where *y* is the phenotypic vector of the quantitative trait (*i.e.*, metabolite), *t* is the number of QTL, β is the vector of regression coefficients, X is the matrix for the main effects including QTLs and covariates (*e.g.*, pericarp color), and ε are the residuals.

The linkage disequilibrium (LD) *r*^2^ and *p*-values were computed using the function *LD* from the R package *genetics* (Warnes, 2002).

## 3 Results

### 3.1 RIL population LC-MS results

A total of 3, 780 compounds were detected in the mature grain of the RIL population, 1, 022 of which were computationally annotated or assigned a metabolite class. Of these compounds, 68% had a CV > 50% (**Supplementary Figure 1A**). For repeatability, only 7% of the metabolites showed a *r* > 0.50, with a maximum *r* = 0.80, while 54% of the compounds had a *r* < 0.20 (**Supplementary Figure 1B**).

### 3.2 Metabolic profile of RIL population based on AM activity groups and grain pigmentation

#### 3.2.1 Multivariate analyses

**Figure 1:**
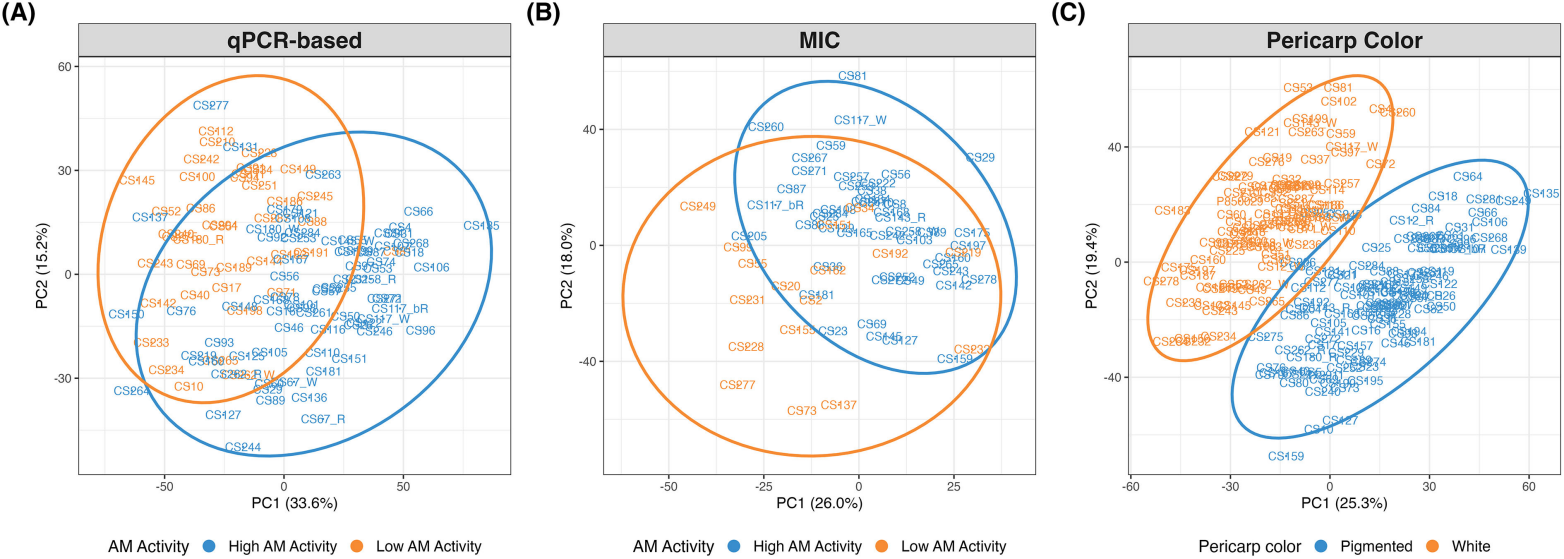
PLS-DA plot for **(A)** *in vitro* qPCR-based and **(B)** MIC AM activity using extreme categories (*i.e*., High and Low AM groups), and **(C)** pericarp color.

The PCA revealed that the first two principal components (PCs) accounted for 52% of the variation (**Supplementary Table 1**); however, no clear separations were observed among the AM activity groups (**Supplementary Figure 2**). To further investigate which metabolites influenced group separations, PLS-DA was performed. When PLS-DA was performed on the entire population (*i.e.*, including High AM, Medium AM, and Low AM RILs), still no clear clustering was observed for either detection method (**Supplementary Figure 2**). Since we are interested in differentiating the extremes, PLS-DA was performed using only the High AM and Low AM groups for each detection method, which resulted in tighter group clustering, albeit with some overlap (**Figure 1**).

**Figure 2:**
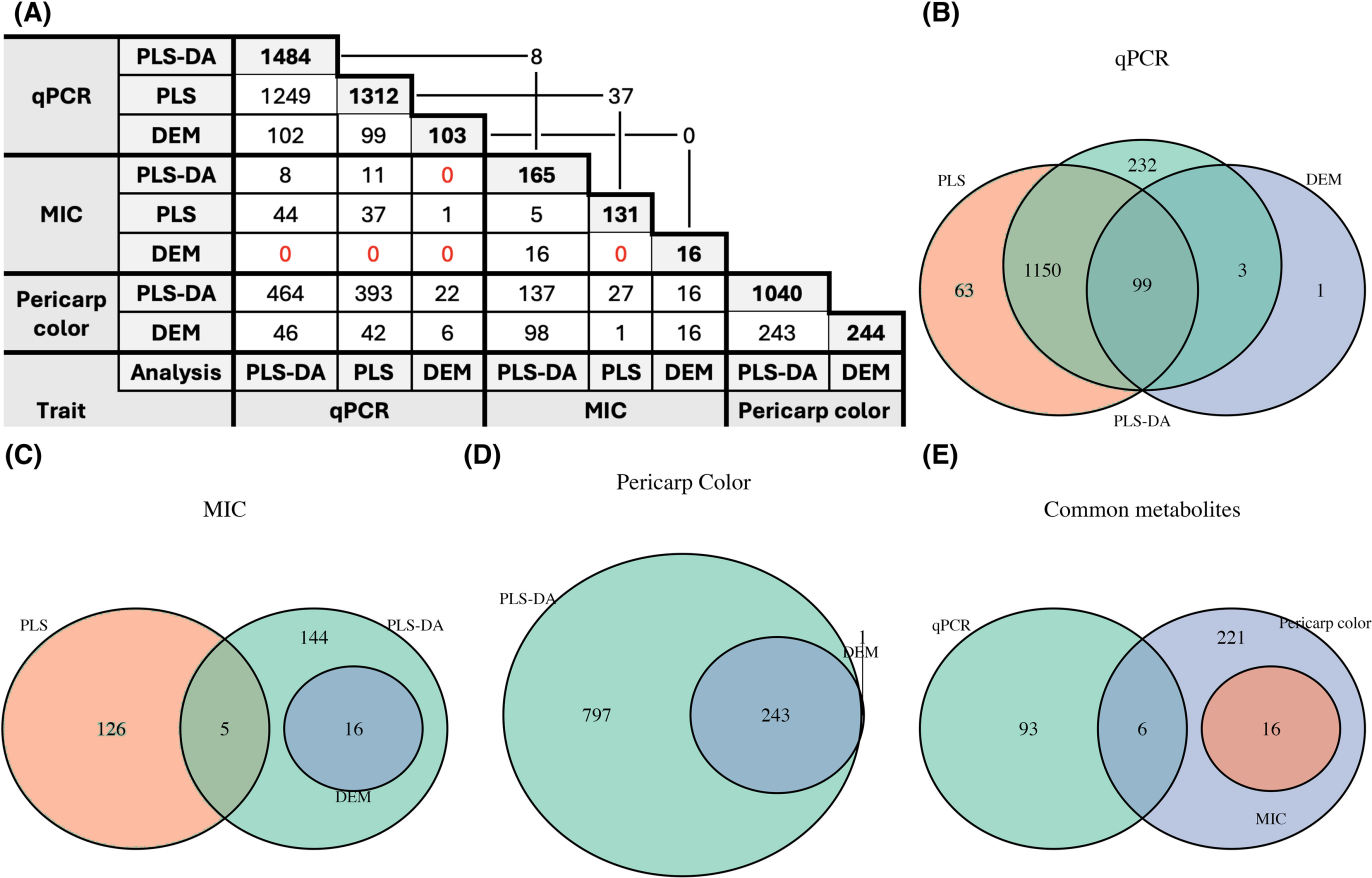
**(A)** Table summarizing intersections of significant metabolites for PLS-DA, PLS and FC analysis (DEM), for AM activity traits (qPCR and MIC) and pericarp color, with the diagonal indicating the significant metabolites for each trait-analysis combination. **(B**–**E)** Venn diagrams showing the number of significant metabolites for each analysis for **(B)** qPCR AM activity, **(C)** MIC AM activity, and **(D)** pericarp color. The common significant metabolites among the three phenotypes are shown in **(E)**.

For the qPCR analysis, the first two PCs explained 49% of the total variation (**Supplementary Table 1**). The 10-fold cross-validation yielded an AUC ≥ 0.80 (**Supplementary Table 2**), indicating strong discriminative performance. Additionally, analysis of variable importance revealed 1, 484 out of the 3, 780 metabolites had a VIP ≥ 1 (**Figure 2A**), all of which were significant (*p*-value< 0.05), suggesting that the separation between High AM and Low AM classes was influenced by a wide range of compounds, likely across multiple metabolic pathways.

Similarly, for the MIC assay, the first two PCs explained 44% of the total metabolic variation (**Supplementary Table 1**), showing compound clustering (**Figure 1B**). However, the 10-fold cross-validation yielded an AUC ≤ 0.60 (**Supplementary Table 2**), indicating weak class separation and model performance. Furthermore, 1, 120 metabolites had a VIP ≥ 1, with only 165 being significant (**Figure 2A**).

Among both sets of significant metabolites, eight were shared across detection methods (**Figure 2A**), indicating that each method captured different aspects of AM activity and, thus, different metabolic profiles. Interestingly, for the qPCR assay, 92% of metabolites with VIP ≥ 1 were more abundant in the Low AM group. The opposite was observed for the MIC, with 72% of the compounds with VIP ≥ 1 having higher abundance in the High AM. However, of the top 100 highest VIP compounds of the MIC assay, 74% were higher in Low AM, suggesting that, although the majority of the metabolites have inhibitory effects, the most influential metabolites in distinguishing AM activity groups were more abundant in the Low AM RILs.

When performing PLS-DA on pericarp color, clear separation was observed between pigmented and white samples (**Figure 1C**), showing very distinct metabolic profiles. The 10-fold cross-validation indicated excellent performance of the model with an AUC > 0.90 (**Supplementary Table 2**). There were 1, 044 compounds with VIP ≥ 1, of which 1, 040 were significant (*p*-value< 0.05) (**Figure 2A**). Interestingly, most compounds (69%) with VIP ≥ 1 were higher in the white pericarp lines; however, the opposite was observed for the top 100 highest VIP compounds, with 88% of them having higher abundance in the pigmented sorghum RILs.

#### 3.2.2 Metabolic pathway enrichment based on AM activity category and pericarp color

**Figure 3:**
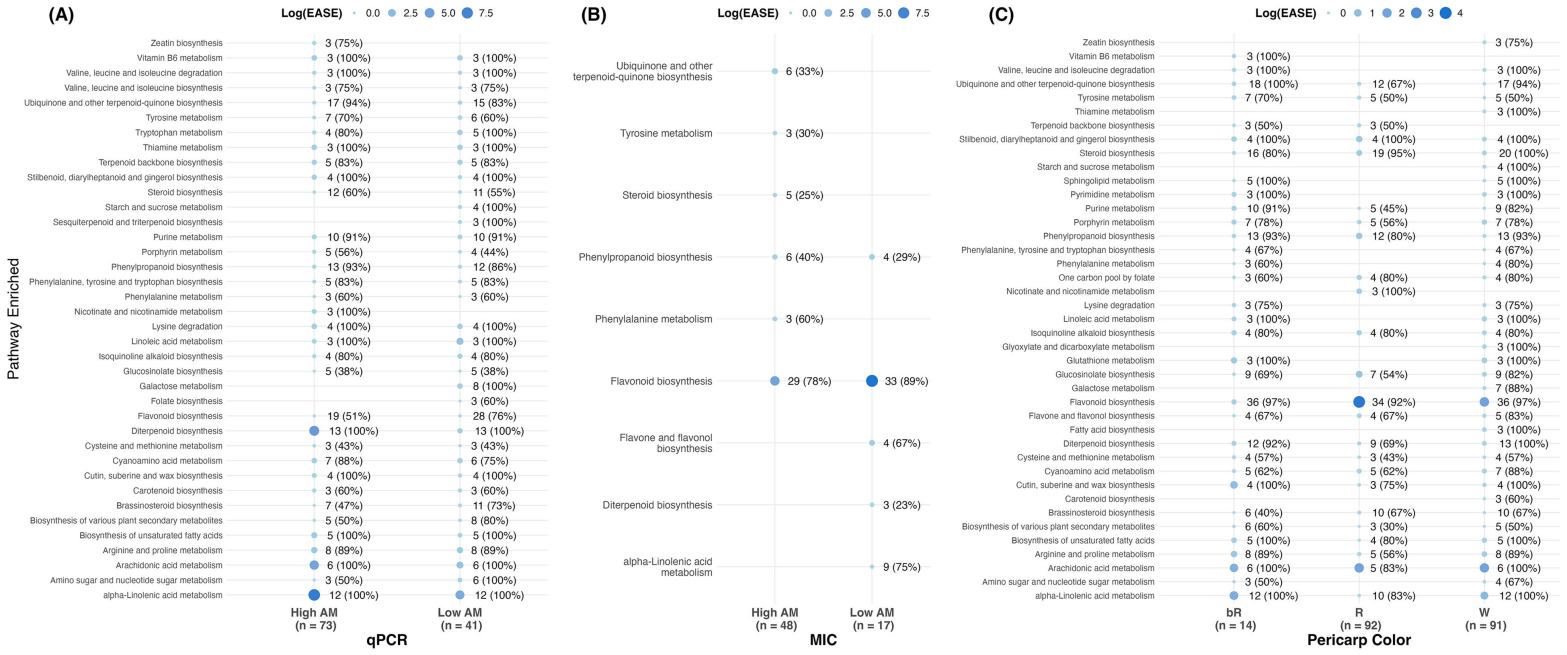
Pathway enrichment analysis listing pathways that showed at least 3 significant compounds for each group relative to the rest of the population for **(A)** qPCR AM activity, **(B)** MIC AM activity, and **(C)** pericarp color (R, red; W, white, and bR, bright red). The numbers next to each point indicate the significant compounds and the percentage of the total compounds that mapped to that pathway.

The RIL population showed widespread enrichment of metabolic pathways as well as group-specific enrichment (**Figure 3**). The metabolic profiles of the extreme groups were compared to those of the rest of the population: High AM versus Medium AM + Low AM, and Low AM versus Medium AM + High AM. The metabolites associated with the qPCR assay were present across a higher number of known metabolic pathway enrichments compared to the metabolites linked to the MIC assay. For the qPCR High AM group (**Figure 3A**), the highest enrichment was observed for alpha-linolenic acid metabolism, diterpenoid biosynthesis, and arachidonic acid metabolism, as denoted by a higher EASE score, which indicates enrichment of a given metabolic pathway in a group *relative* to the rest of the population. The low AM group also showed enrichment for alpha-linolenic acid metabolism and diterpenoid biosynthesis, albeit to a lesser extent. The MIC exhibited high enrichment in flavonoid biosynthesis (**Figure 3B**) across both groups, with Low AM having higher log_10_(EASE) than High AM. These enrichments were confirmed by comparing only the extreme group (without the intermediate RILs) (**Supplementary Figure 3**).

Pericarp color exhibited widespread enrichment across multiple pathways (**Figure 3C**). Flavonoid biosynthesis was the most enriched pathway, followed by arachidonic acid metabolism and alpha-linolenic acid metabolism.

#### 3.2.3 Differentially expressed metabolites in extreme categories

**Figure 4:**
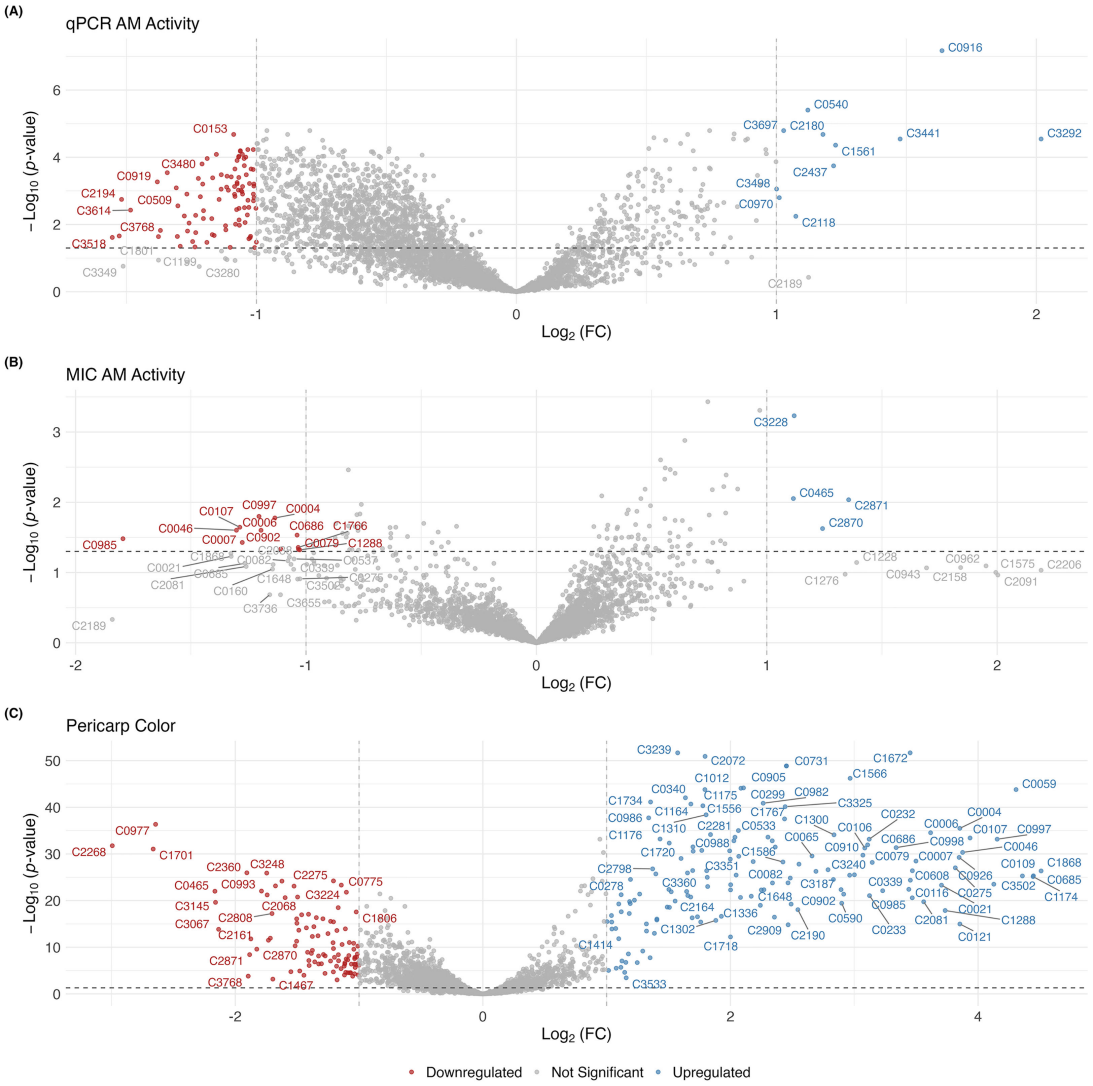
Volcano plots capture differentially expressed metabolites for **(A)** *in vitro* qPCR-based AM activity, **(B)** MIC AM activity, and **(C)** pericarp color. In **(A)** and **(B)**, metabolites in blue are the significant upregulated compounds in the High AM groups, while the red metabolites represent downregulated metabolites. For pericarp color **(C)**, blue metabolites are the significant compounds upregulated in the pigmented RILs.

Fold-change (FC) analysis was performed to identify DEMs between extreme groups of RILs. When examining FC between pigmented and white RILs, there were 244 significant DEMs (**Figure 2A**), with 58% of the compounds being upregulated in the pigmented sorghum (**Figure 4C**). Of those metabolites, 243 were also identified by the PLS-DA model (**Figure 2D**, **Supplementary Table 3**).

When examining the qPCR categories, 103 significant compounds with an absolute log_2_(FC) ≥ 1 (**Figure 2A**) were detected. Of these 103 metabolites, the majority were downregulated (89%) in the High AM group, with only 11% of compounds being upregulated (**Figure 4A**). Only six of the 103 DEMs were differentially expressed among pericarp color classes (**Figure 2A**), indicating that this AM activity was not substantially driven by pigment-related compounds. Integrating differential abundance (|log_2_(FC)| ≥ 1) with multivariate importance metrics (PLS-DA VIP ≥ 1 and PLS VIP ≥ 1) identified 99 metabolites associated with qPCR-based AM activity (**Figure 2B**) that exhibited a repeatability range of 0.04–0.68 (mean = 0.36), with only nine of these metabolites being upregulated in the High AM group (**Supplementary Table 3**).

For the MIC categories, only 16 significant DEMs were identified, of which 75% were downregulated in the High AM (**Figure 4B**). No DEMs were shared between the two AM methods (**Figure 2A**). All 16 significant compounds were shared with pericarp color, indicating that the compounds associated with MIC-based AM activity were linked to pigmentation (**Figure 2A**). When integrating the DEMs with multivariate metrics, no metabolites met all criteria (**Figure 2C**). However, when considering only analyses that examined AM classes (*i.e.*, PLS-DA and DEM), 16 common metabolites were identified (**Figure 2C**), and they exhibited a repeatability range from 0.32–0.77 (mean = 0.55), with all but four metabolites being downregulated in the High AM group (**Supplementary Table 3**).

**Figure 5:**
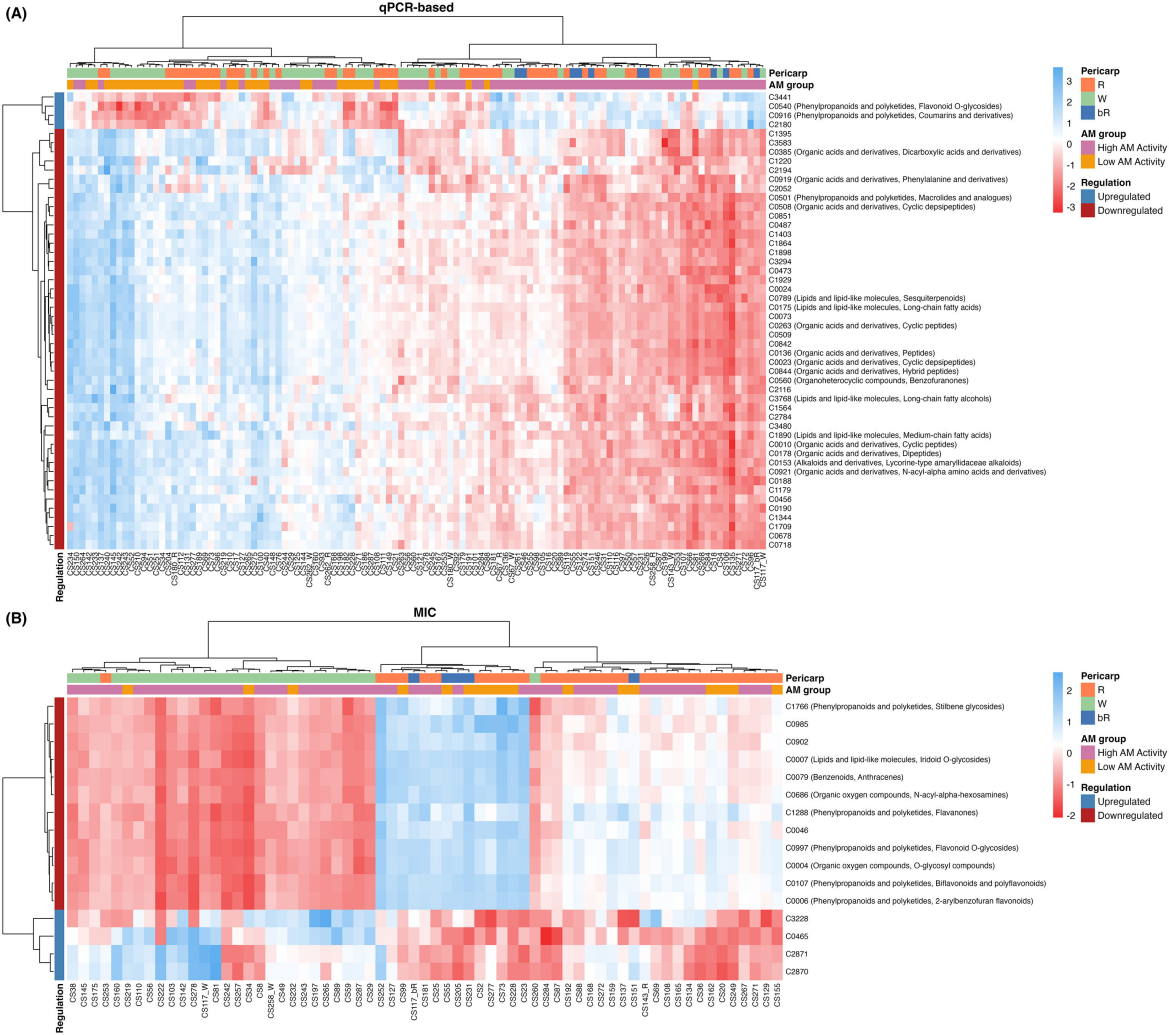
Heatmaps of key metabolites (vertical labels) for **(A)** qPCR-based AM activity and **(B)** MIC AM activity highlight regulation patterns with respect to pericarp color and metabolite regulation across sorghum recombinant inbred lines (horizontal labels).

In the heatmaps showing the most influential compounds for each AM activity assay (**Figure 5**), two major clusters of metabolites with opposite behaviors were observed. One cluster contained only four and five compounds that respectively matched the upregulated compounds for the qPCR and MIC. The qPCR method better discriminated RILs by AM groups (**Figure 5A**), with two distinct groups characterized by contrasting abundances of metabolite clusters. The largest cluster of metabolites was downregulated and mostly annotated as lipids and organic acids. Conversely, the MIC clustered the RILs predominantly by pericarp color (**Figure 5B**), as expected, since these metabolites were among the most influential in pericarp color discrimination (**Figure 2E**). Five of the 12 downregulated metabolites were annotated as phenylpropanoid compounds.

### 3.3 Variable selection for biomarker and QTL mapping

To identify metabolites that could be used as biomarkers for AM activity, variable selection through sparse (s)PLS-DA and (s)PLS was performed. For the MIC assay, both analyses revealed that the majority of compounds were required to predict the AM activity, indicating that MIC results were noisy and not well-suited for metabolomics-based biomarker discovery (**Supplementary Table 4**), which is consistent with the PCA results. Therefore, the qPCR-based AM activity results were used for biomarker discovery and later QTL mapping.

The sPLS-DA identified 17 compounds that were required to discriminate between groups, with a 10-fold cross-validation yielding an AUC > 0.85 (**Supplementary Table 5**). On the other hand, the sPLS identified 36 compounds. However, the metrics for PC2 showed weak performance (Q^2^ = –0.10 and R^2^ = 0.01) (**Supplementary Table 5**), indicating that a 1-component model (*i.e.*, only using PC1 from sPLS) was most appropriate, with only 10 compounds needed.

Of the metabolites selected from each model, and based on compound stability (**Supplementary Table 6**), 12 compounds were the most reliable (stability > 0.7) in discriminating AM groups (PLS-DA, seven) and predicting qPCR-based AM activity (PLS, nine), with four of those compounds being important in both. Notably, metabolites C0153 (downregulated) and C0916 (upregulated) were selected across all cross-validation folds and repeats (*i.e.*, stability = 1) in both sPLS-DA and sPLS. Their consistent selection across folds suggests they capture main biochemical differences between AM groups, rather than model- or dataset-specific effects, playing a key role in AM activity and its associated metabolic profile. More information on these two specific metabolites can be found in **Supplementary Table 7**.

### 3.4 Metabolite annotation

Given that only five of the 12 selected metabolites (*i.e.*, C0010, C0024, C0153, C0540, C0560, C0916, C1249, C1585, C1864, C2295, C2431, and C3480) were annotated using spectral matching and computational interpretation, they were manually evaluated by molecular weight searching against a small molecular structure library composed of biologically-linked PubChem compounds (Broeckling, 2026). Additionally, natural pressure of sorghum grain mold was occurring in the tested environment, and the RIL population was segregating for grain mold resistance. Thus, because *Fusarium* is the main genus in the grain mold complex, and because some metabolites had no significant matches against known *Sorghum* metabolites, they were also searched against *Fusarium*.

Manual examination of the selected metabolites revealed that they likely represent 13 different compounds. It was found that compounds C0540 and C1585 likely represent different adducts of the same compound (the C0540 signal was used), and was annotated as bis-ferulamidobutane (a hydroxycinnamic acid), a conjugate of putrescine and two ferulic acid molecules (**Supplementary Table 8**). Moreover, compounds C0153 and C1864 represented, in fact, two different compounds each, C0153a-b and C1864a-b (**Supplementary Table 7**), which were clustered together by RAMClustR due to their highly similar retention time and signal intensity correlation. Compounds C0153b (a polyketide) and C0560 (a benzoic acid) are known *Fusarium* metabolites and mapped to *Fusarium* taxonomy, indicating they are likely pathogen-derived rather than sorghum metabolites (**Supplementary Table 8**). Both compounds contain chlorine, and their distinct isotopic signature in the mass spectrum supports the assigned molecular formulas. Compound C1864a, a naphthopyran, mapped to both *Fusarium* and sorghum. Meanwhile, for C1864b, no biological molecular formula could be found that matches this compound’s inferred molecular weight.

Of the remaining metabolites, C2431 was consistent with the compound N’-Methylniphimycin, which was reported in *Streptomyces* (Ivanova et al., 1998), but lacked evidence of plant occurrence. C2295 was classified as elliptinone, a type of naphthoquinone, with only a few records of plant occurrence, particularly in the genus *Diospyros*, and known to be a bioactive compound with antimicrobial effects (Higa et al., 2002; Kaewbumrung and Panichayupakaranant, 2012). For C1249, the closest structure identified was edgeworin, a hydroxycoumarin. There is little information about this compound, which was identified in the *Edgeworthia* genus and found to have anti-inflammatory and other beneficial effects (Hu et al., 2008).

Compounds C0010, C0024 and C0153a were not taxonomically linked to plants. However, C0024 and C0153a were identified as a cardiolipin and a fatty acyl glycoside, respectively, classes of lipids that can be found in plants (Schlame and Ren, 2009; Michaelson et al., 2016). Additionally, C0010, an alkaloid, has evidence of being a plant metabolite (Zhang et al., 2020). Compound C3480 was identified as 10-hydroxyphaeophorbide, a chlorophyll breakdown product, known to occur in plants (Mohn et al., 2009).

### 3.5 Loci associated with the most significant metabolites

QTL mapping was performed for the 13 metabolites. No peaks were detected for five of 13 metabolites (**Supplementary Figure 4**). A list of peaks for the remaining eight compounds is in **Table 1**. Because the mapping for AM activity in Conti et al. (2026) was influenced by the pericarp color QTL which showed an extremely high LOD score (56.6), the effect of this QTL was tested for each of the 13 selected metabolites. All compounds except two (C0540 and C1249) showed significant differences (*p*-value< 0.05) in relative abundance between pericarp colors (**Supplementary Figure 5**). QTL mapping was repeated using pericarp color as an interactive covariate, yielding new peaks (**Supplementary Figure 6**, **Table 1**).

**Table 1:** Table summarizing QTL mapping results for the selected metabolites, showing chromosome (Chr) and position (Pos) in Mb of the peak, LOD score and marker name for models with and without pericarp color as a covariate.

Metabolite	Without covariate				With pericarp color covariate				LD *r*^2^ AM trait 01_25853452
	Chr	Pos(Mb)	LOD	Marker	Chr	Pos(Mb)	LOD	Marker	
C0010 (↓)	–	–	–	–	2	61.23	3.10	02_61232231	
					5	7.36	3.59	05_7359215	
C0024 (↓)	–	–	–	–	1	25.1	3.05	01_25095211	0.93
					5	7.36	3.62	05_7359215	
					10	54.27	3.63	10_54273941	
C0153a (↓)	–	–	–	–	–	–	–	–	
C0153b (↓)	1	69.59	6.87	01_69588043	1	23.06	3.96	01_23064345	0.81
	4	58.60	3.08	04_58595900	4	63.34	3.15	04_63341727	
					10	54.27	3.58	10_54273941	
C0540 (↑)	1	25.62	4.12	01_25619342	1	25.62	4.26	01_25619342	1
	2	61.23	4.54	02_61232231	2	61.23	5.90	02_61232231	
	5	68.88	3.03	05_68877926	5	68.88	4.56	05_68877926	
C0560 (↓)	1	24.81	3.31	01_24808958	1	24.81	3.65	01_24808958	0.93
	2	11.51	3.56	02_11505474	2	11.51	3.54	02_11505474	
					2	61.15^*^	3.29	02_61154136	
C0916 (↑)	1	25.62	3.07	01_25619342	1	25.62	3.29	01_25619342	1
	2	66.61	3.81	02_66605824	2	66.61	3.98	02_66605824	
	2	61.15^*^	3.26	02_61154136	2	61.15^*^	3.68	02_61154136	
C1249 (↓)	4	56.05	4.15	04_56051636	3	67.38	3.20	03_67384432	
	7	53.17	9.61	07_53170233	4	56.05	4.09	04_56051636	
					7	54.55	9.87	07_54554396	
C1864a (↓)	–	–	–	–	–	–	–	–	
C1864b (↓)	1	61.73	4.01	01_61728936	5	69.30	3.56	05_69295030	
C2295 (↓)	7	54.55	7.60	07_54554396	3	67.21	3.17	03_67205526	
					7	54.55	7.51	07_54554396	
C2431 (↓)	1	64.43	3.04	01_64433857	–	–	–	–	0.01
C3480 (↓)	–	–	–	–	–	–	–	–	

^*^ Second peak. If applicable, the LD *r^2^* with the previously identified QTL for qPCR AM activity (marker 01_25853452) is shown.

Four metabolites (C0153a, C1864a, C2431, and C3480) still showed no peaks when pericarp color was used as a covariate, indicating that their abundance is influenced by multiple small-effect loci. Metabolite C2431 initially mapped to chromosome (chr) 1, but this peak fell within the pericarp color QTL region and disappeared when pericarp color was added as a model covariate. The remaining nine metabolites mapped to multiple genomic regions that commonly overlapped, except for C1864b, which mapped to a single locus.

Interestingly, the two compounds selected from PLS-DA PC2, C1249 and C2295, mapped to a region on chr07 (LODs > 7) that co-located with the height QTL detected for this population (Conti et al., 2026), ~6 Mb away from the *dw3* gene (*Sobic.007G163800*) (Multani et al., 2003). Further, both compounds mapped to a QTL on chr03 linked to anthracnose resistance (Conti et al., 2026). Additionally, their absolute FC values between AM classes were< 0.60 (**Supplementary Table 8**). This suggests that PC2 might have captured variation related to plant architecture or growth rather than AM activity. Thus, although these metabolites were associated with AM activity, they were not key drivers of the trait.

**Figure 6:**
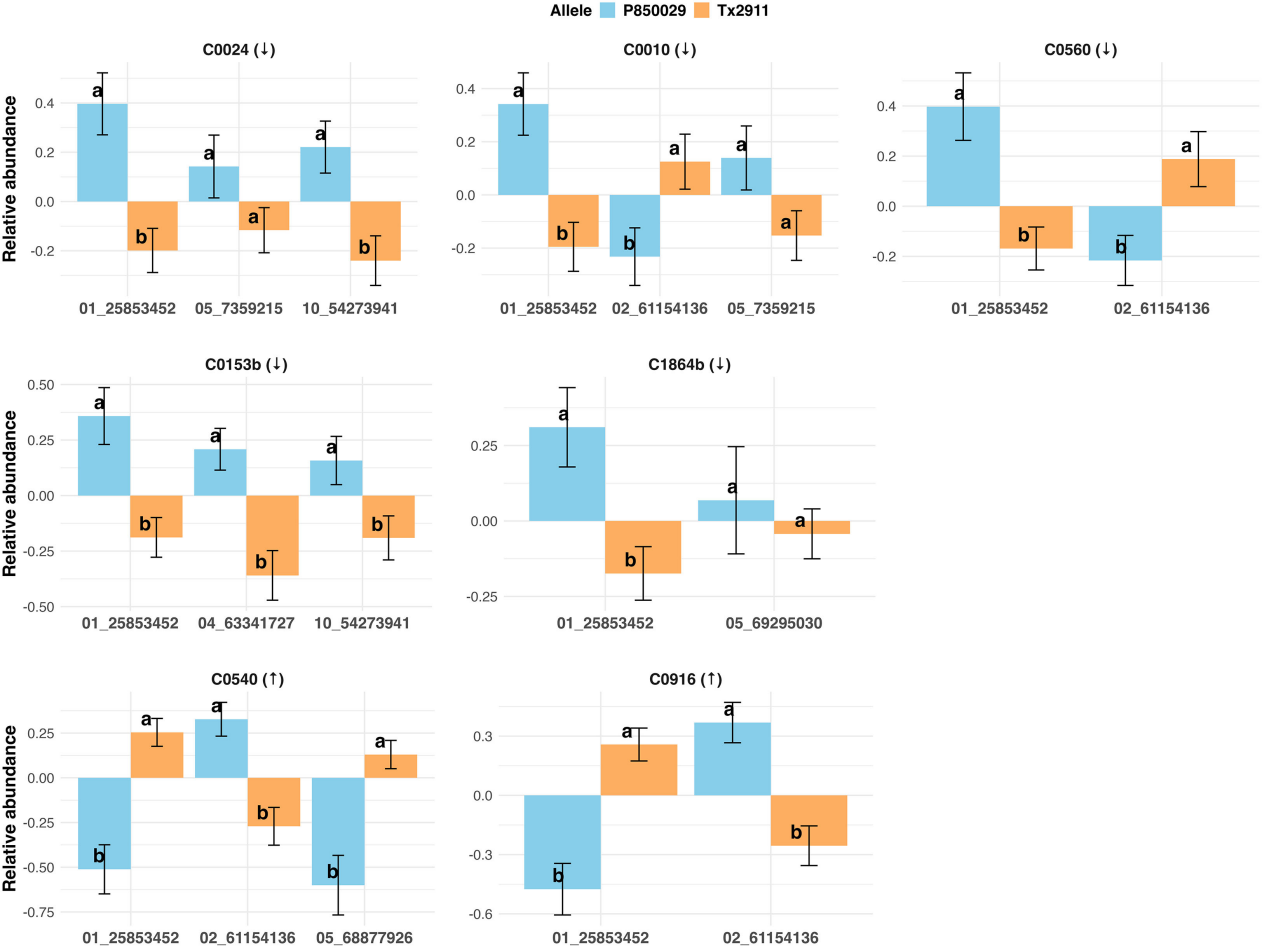
Barplots show the scaled intensities for each allele (Tx2911 and P850029) of the markers of interest, for seven metabolites, five with lower (↓) and two with higher (↑) relative abundance in the High AM activity group. Letters of significance denote statistical differences between alleles based on the LSD test (*p*-value< 0.05).

**Figure 7:**
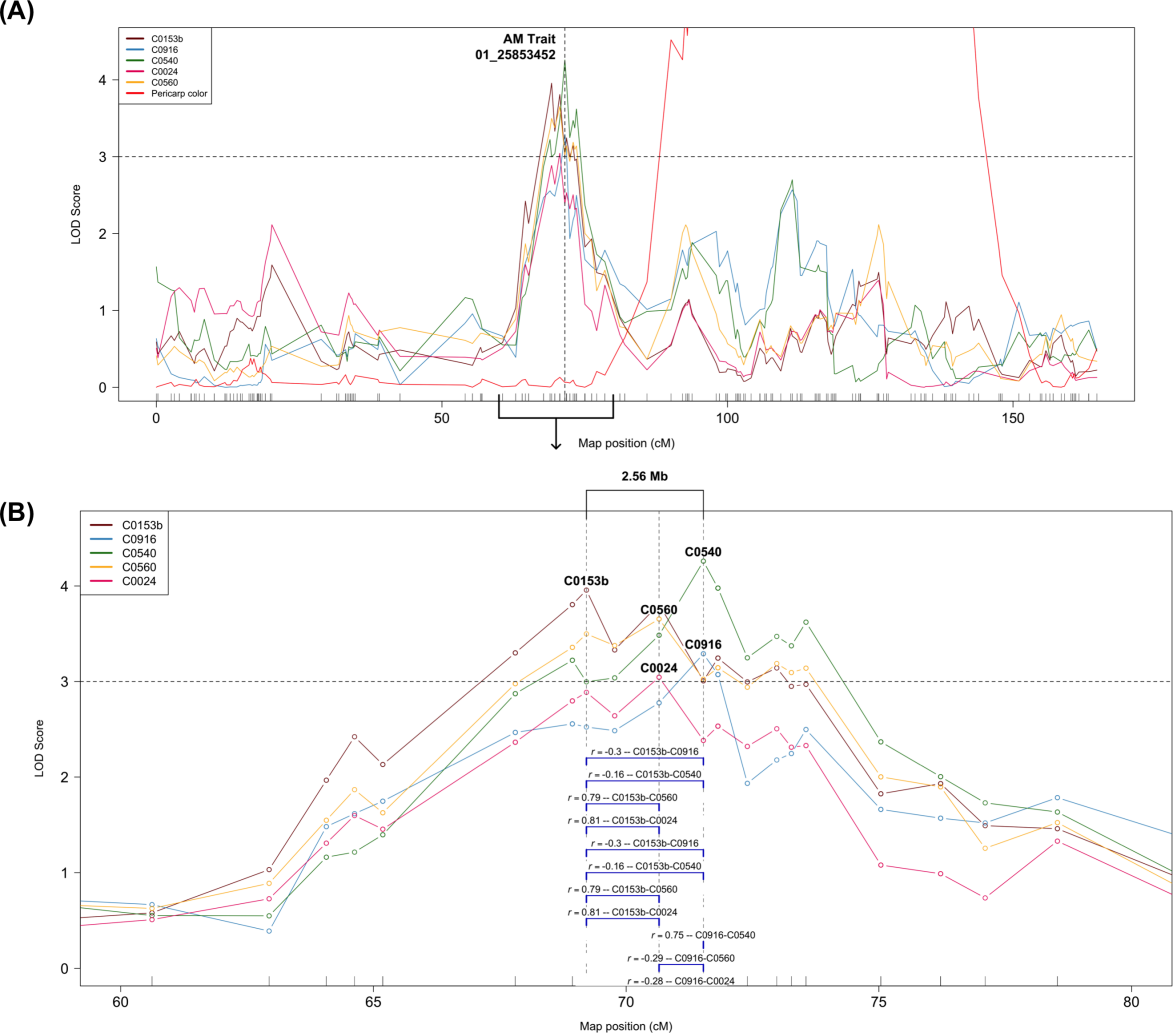
Manhattan plots of chromosome 1 showing **(A)** five metabolites with pericarp color as an interactive covariate and the pericarp color QTL on the full chromosome length, with the vertical dashed line indicating the position of the AM trait QTL. **(B)** Magnification of the chr01 region linked to AM metabolites highlights the region’s physical distance and the Pearson correlation coefficient for each metabolite pair based on relative abundance. The horizontal dashed line indicates the LOD score threshold for significance.

Metabolites C0010, C0024, C0153b, C0540, C0560, and C0916 each mapped to two or three significant peaks positioned on chr01, chr02, chr04, chr05 and chr10 (**Table 1**). C0153b peak on chr01 without pericarp color covariate was at 69.59 Mb, in strong LD with the pericarp color QTL (*r*^2^ = 0.70). When pericarp color was included in the model, a new peak was detected at 23.06 Mb. Interestingly, the favorable allele for AM activity at the chr02 locus was the only one that came from P850029. The favorable allele for AM activity at the remaining loci came from Tx2911 (**Figure 6**). Five metabolites showed a peak on chr01 (**Figure 7A**). Of these, two (C0540 and C0916) had higher abundance in the High AM group and were in complete linkage with the previously identified QTL for qPCR-based AM activity (Conti et al., 2026). The remaining three metabolites (C0024, C0153b, and C0560) had lower abundance in the High AM RILs and were in LD (*r*^2^ > 0.81, **Table 1**) with this same QTL. Metabolites C0540 and C0916 also mapped to a secondary QTL on chr02 at ~61 Mb (**Table 1**, **Supplementary Figure 7**), where metabolites C0010 and C0560 also showed a small peak (**Table 1**).

Based on the allele-effect analysis (**Figure 6**), the peak on chr05 at 7.36 Mb appears to have little effect, with no significant difference in metabolite abundance between alleles (C0010 and C0024). However, the other peak on chr05 at ~69 Mb identified for C0540 and C1864b showed a large difference in metabolite abundance between alleles, but only for C0540 (**Figure 6**). Additionally, the QTL observed on chr10 for C0024 and C0153b colocalized with a TGW QTL previously identified in this population (Conti et al., 2026), showing differences in abundance between alleles.

For the large chr01 QTL, because the peaks for the five metabolites were within 2.31 cM (and just 2.56 Mb), the selected marker for analysis was 01_25853452 (AM trait marker) (**Figure 7**). There were significant differences in the relative abundance between alleles for each metabolite, even for C0010 and C1864b, which showed no significant LOD score at this locus (**Figure 6**). Furthermore, all correlations among relative abundance for the five metabolites mapping to chr01 were significant (*p*-value< 0.05) (**Figure 7B**). Metabolites with higher abundance in the High AM group were positively correlated with each other, as were those with lower abundance. Additionally, pairwise correlations between higher and lower-abundance metabolites were negative (**Figure 7B**).

Based on allelic effects and the two- and three-loci models analyzed (**Supplementary Figure 8**), it was observed that the locus on chr01 is a major-effect QTL for the seven metabolites under consideration. A favorable allele at this chr01 locus largely dictated the metabolite abundance and AM activity, with a PVE for each metabolite from 7-22% (**Supplementary Table 9**). Interestingly, this locus also explained a considerable proportion of the variance in metabolite abundance for the four compounds (C0153a, C1864a, C3480, and C2431) with no significant QTL (PVE = 5.1–10.8%, **Supplementary Table 9**).

## 4 Discussion

Sorghum grain samples for the RIL population Tx9211/P850029, which were harvested at physiological maturity and stored at room temperature until use, were ground into flour to perform untargeted metabolomics, *in vitro* digestion (qPCR assay) and phenolic extraction (MIC assay). These datasets were then used to link differentially expressed metabolites with AM activity and the putative loci underpinning this trait. A comprehensive profile of constitutive (pre-existing) and environmentally-induced (during grain fill stages) metabolites was obtained. By integrating these metabolite profiles with AM activity classification derived from the qPCR and MIC assays, associations between grain chemical composition and inhibitory effects against *C. perfringens* were identified.

### 4.1 Pathway enrichment

Pathway mapping was performed using the metabolic reference model *Zea mays* from the KEGG global metabolic network as a proxy for sorghum metabolism, as it was the closest available library at the time of analysis. Thus, species-specific differences in secondary metabolism could influence the results. Nevertheless, maize provided a reasonable proxy within the Poaceae family, and the observed enrichment patterns were consistent with sorghum metabolism.

The non-tannin RILs exhibited a rich grain metabolomic profile among the 3, 780 compounds under investigation. It was demonstrated that RILs differing in AM activity also exhibited differences in their metabolic profiles (**Figures 5A, B**). The inconsistencies found between AM detection methods were somewhat expected since no correlation was observed between the two AM phenotypes (Conti et al., 2026). Alpha-linolenic acid metabolism was the most enriched pathway for the qPCR assay classification, whereas for the MIC it was flavonoid biosynthesis, as it was for pericarp color (Dykes et al., 2009; Makanjuola et al., 2018; Moritz et al., 2023).

Alpha-linolenic acid is a precursor of jasmonic acid (JA), a plant hormone involved in signaling and responses to both abiotic and biotic stresses (Wasternack and Song, 2017). Diterpenoid biosynthesis was another pathway that showed enrichment for qPCR AM activity. Diterpenoids are plant defense secondary metabolites known to have AM effects, acting as local AM phytoalexins in monocot crops such as rice (*Oryza sative*) and maize (Block et al., 2018; Murphy and Zerbe, 2020). These compounds have been reported to inhibit pathogens, including *Magnaporthe oryzae*, the causal agent of rice blast, and *Fusarium verticillioides* and *F. graminearum*, maize fungal pathogens (Block et al., 2018; Murphy and Zerbe, 2020). Another enriched pathway for qPCR was arachidonic acid metabolism. Arachidonic acid is a polyunsaturated fatty acid produced downstream of linoleic acid and involved in JA metabolism. It was reported to act as a signaling molecule in plant defense, effectively modulating stress-responsive transcriptional networks, and shown to reduce susceptibility to *Botrytis cinerea* infection in tomato (*Solanum lycopersicum*) (Savchenko et al., 2010). Health-promoting properties of arachidonic acid, including for cardiovascular diseases, cancers, and inflammatory diseases, have also been reported in humans (Wang et al., 2021).

The enrichment of the flavonoid biosynthesis pathways for the MIC and pericarp color highlights the central role of phenylpropanoid-derived metabolites in sorghum grain. Flavonoids are the largest group of phenolic compounds and contain many pigment-associated metabolites (Dykes et al., 2009; Awika, 2017; de Morais Cardoso et al., 2017), which is why they were enriched in the pericarp color analysis. The MIC assay relied on acetone extraction, a widely used solvent for phenolic extractions in sorghum grain, which recovers soluble secondary metabolites (Herald et al., 2012; Rhodes et al., 2017; Shields et al., 2021). Thus, the enrichment of flavonoid biosynthesis suggests that constitutive phenolic compounds dominated the extractable metabolites that contributed to the variation observed for AM activity.

### 4.2 Multivariate analyses and DEM

Multivariate and relative abundance analyses comparing extreme categories revealed that the majority of compounds associated with AM activity were downregulated in the High AM group (**Figure 4**), indicating that many metabolites are linked to susceptibility rather than resistance. The few upregulated compounds could be specialized metabolites that are driving the resistance.

Specifically, the qPCR phenotype showed a broader metabolite shift than the MIC, consistently detecting a larger number of significant metabolites associated with AM activity across all analyses (**Figure 2**), indicating a strong metabolic signature. This was consistent with the observed large number of pathways enriched. Meanwhile, the MIC showed weaker metabolic differentiation between AM groups, suggesting overlapping metabolite profiles, as observed with the pathway enrichment analysis with flavonoid biosynthesis being enriched in both groups. These results, along with the poor performance of the PLS-DA and PLS models (**Supplementary Table 2**), could be attributed to the unbalanced data, given the lower number of Low AM RILs. These findings were confirmed by a weak metabolic signal for the MIC phenotype, as indicated by the poor predictability of sPLS regression and sPLS-DA (**Supplementary Tables 2**, **S4**). There were minor metabolic differences related to the minimum concentration of sorghum extract needed to inhibit the growth of *C. perfringens*. This finding could be attributed to the extraction process since it only captured acetone-soluble compounds (Herald et al., 2012). The low overlap of metabolites between the two AM detection methods indicated that each method detected different AM profiles, suggesting that they may be measuring different aspects of AM activity.

Investigation of pericarp color showed that RILs with white pericarp exhibited broad metabolic differences, as indicated by over 1, 000 metabolites that showed higher abundance in those individuals. These widespread changes likely reflect shifts across multiple metabolic pathways, rather than pathways directly involving pigmentation. In contrast, the 100 strongest discriminating metabolites were predominantly enriched in pigmented RILs (*i.e.*, RILs with red pericarp color), suggesting pathway-specific variation associated with pigment biosynthesis.

The heatmaps also showed that there were High AM RILs that exhibited an opposite metabolic profile/behavior compared to the rest of the group. This is because the PLS-DA VIP scores detected the metabolites that best separated the *average* High AM from the *average* Low AM. However, there could be individual RILs within each group that had a different pattern of enrichment from the average. This indicates that alternative biochemical pathways may exist to achieve the same level of AM activity, which could be attributed to variations in the genetic background.

### 4.3 Metabolite selection and classification

Although many compounds were associated with AM activity, variable selection analyses revealed that 12 metabolites (later identified as 13) were sufficient to accurately predict qPCR AM effects, indicating that the phenotype has relatively small underlying metabolic control. For these metabolites, we assigned the following annotations manually, based on molecular weight consistency with metabolites that could reasonably be expected to be produced by sorghum or *Fusarium*, which is known to occur under field conditions.

Of the two compounds with higher relative abundance in the High AM RILs, C0916 was identified as lysoPA(16:0)(**Supplementary Table 8**), a lipid molecule containing a palmitoleic acid chain, and a known metabolic product of and taxonomically linked (Broeckling, 2026) both sorghum and *Fusarium*. Phosphatidic acid is involved in lipid metabolism, membrane structure, and signaling in response to abiotic and biotic stresses (Yang and Frohman, 2012; Yao et al., 2025). The accumulation of phosphatidic acid in the High AM RILs may reflect a defense response to the natural pathogen pressure and abiotic stress present in the field. It enhances membrane integrity and can signal pathways to activate defensive gene expression and hormone production, such as JA (Wasternack and Song, 2017). This aligns with the observed enrichment of alpha-linolenic acid metabolism and arachidonic acid metabolism. Meanwhile, C0540 was identified as a ferulic acid amide, bis-ferulamidobutane. This hydroxycinnamic acid has been reported in corn barn to provide high anti-inflammatory activity and other health benefits (Kim et al., 2012). Additionally, it was documented that ferulic acid amides, such as bis-ferulamidobutane, are incorporated into the lignin polymer in maize seed coats, thereby enhancing cell wall integrity and strength (Del Río et al., 2018). The higher abundance of this compound in the High AM RILs could reflect a stress response that reinforces plant tissue against pathogens such as *Fusarium*.

C0540 and C0916 are constitutively expressed because they are involved in fundamental plant functions, such as membrane structure, lipid metabolism, and cell wall integrity. Thus, the High AM RILs could exhibit a constitutive baseline that is inherently higher for these compounds (whose levels can be further increased under stress), thereby contributing to enhanced AM activity.

Of the metabolites with lower abundance in the High AM group, C0153b and C0560 are consistent with *Fusarium* compounds. C0560 was identified as radicicol, a mycotoxin known to inhibit the growth of other microbes and to suppress the plant’s immune response to stresses by targeting the heat shock protein Hsp90 (Schulte et al., 1999; Xu et al., 2012). Similarly, C0153b, which was identified as pochonin N, is a toxic polyketide produced by *Fusarium* reported to also have growth inhibitory activity (Yang et al., 2011). The lower abundance of these metabolites in the High AM lines aligns with the previously described roles of C0540 and C0916 in plant defense.

Of the remaining compounds with lower abundance in the High AM RILs, C0010, C0024, and C0153a lacked any reported taxonomic links using *pubchem.bio*, but there is evidence of their occurrence in plants (Zhang et al., 2020). The closest structure consistent with the molecular weight for C0010 was the alkaloid CHEMBL4636434. There is limited information about this compound, but evidence suggests it is a plant metabolite with cytotoxic effects (Zhang et al., 2020). C0024 was identified as a cardiolipin, CL(8:0/8:0/a-13:0/18:2(9Z, 11Z)), a type of phospholipid found in mitochondrial membranes, which can act as a signaling molecule (Schlame and Ren, 2009). C0153a was also identified as a lipid, HexCer 8:1;2O/12:0, a particular species of sphingolipid which is known to occur in plants (Michaelson et al., 2016). They are essential components of the plasma membrane and other endomembrane of plant cells and are also involved in stress responses (Markham et al., 2006). A lower concentration of these compounds, C0024 and C0153a, in the High AM lines suggests changes in plant lipid composition, and a reduced need for cardiolipins and glycoside sphingolipids could indicate that defense mechanisms are already activated and/or other lipid types are prioritized (*e.g.*, C0916). Similarly, the lower abundance of C2295 in High AM lines, despite its antimicrobial properties (Higa et al., 2002; Kaewbumrung and Panichayupakaranant, 2012), could be due to a lower overall constitutive level and to other defense-related pathways already active, which might be more effective or less energy-consuming. In the same way, the lower level of C1249 in the High AM group, despite its known bioactivity (Hu et al., 2008), could indicate that the plant is prioritizing other products of the phenylpropanoid pathway, such as C0540, resulting in lower concentrations of this compound. This also indicates that, although several beneficial metabolites were present in the grain, some were less relevant contributors to the AM activity in the context of the qPCR, which could mean they were unstable, less bioactive, not bioaccessible, or degraded during the digestion process, reducing or minimizing their effect against *C. perfringens* (Dima et al., 2020).

C1864a, a naphthopyran, mapped to both sorghum and *Fusarium*; however, most of the evidence suggests it is likely a fungal metabolite (Young et al., 2009; Nicholson et al., 2015), as were C0153b and C0560. Thus, its lower abundance in High AM RILs could be due to the lower overall grain mold observed in these lines (Conti et al., 2026).

The compound C2431, which was less abundant in High AM lines, was found to be associated with chlorophyll breakdown (Pružinská et al., 2003). This could indicate that High AM RILs had healthier tissue or slower senescence, resulting in lower levels of chlorophyll degradation products.

### 4.4 QTL mapping and loci discovery

The presence of 2–3 significant QTL for six of the seven compounds associated with AM activity indicates oligogenic control of metabolite abundance. Interestingly, *Fusarium* metabolites C0153b and C0560 mapped to the same region on chr01 as sorghum metabolites C0024, C0540, and C0916, and the AM trait (Conti et al., 2026), with all peaks within a 2.56 Mb region (**Figure 7B**). The favorable allele at this putatively pleiotropic QTL largely determined AM activity (PVE = 7.4–22.8%) (**Figure 6**; **Supplementary Figure S8**; **Supplementary Table 9**), as reported by Conti et al. (2026). Each allele at this locus has an opposite effect on higher and lower-abundance metabolites, with the Tx2911 allele having a favorable effect on the AM activity (**Figure 6**).

Metabolite C0916 was linked to both sorghum and *Fusarium* (Broeckling, 2026), bis-ferulamidobutane (C0540) is a conjugate of molecules that fungi can produce (Numpaque et al., 2016), and cardiolipins (C0024) can also be produced by *Fusarium* (Sun et al., 2024). Therefore, it could be thought that these compounds mapped to the same region as the *Fusarium* compounds (C0153b and C0560) because they are, in fact, derived from the fungus. However, the stronger pairwise correlations observed between metabolite abundances and qPCR AM activity than with SGM pressure suggest that these metabolites are likely sorghum metabolites (**Supplementary Figure 9**).

The consistent mapping of these metabolites to the previously reported AM locus on chr01 suggests this genomic region might harbor a key regulatory gene(s) involved in a constitutive and defense-related pathway (*e.g.*, phenylpropanoids or lipids) or a step shared across pathways, affecting or regulating loci on other chromosomes, and acting as a metabolic switch to coordinate opposing biosynthetic pathways (Grotewold, 2005). More research is warranted to confirm whether the AM trait is linked to constitutively produced metabolites, phytoalexin-dependent, or a combination of both. Gathering grain samples from healthy plants in absence of disease and inoculated plants, likely in a controlled environment, would help answer this question.

## 5 Conclusion

The rise in antibiotic resistance poses a significant challenge to the poultry industry, particularly in controlling *C. perfringens*. Alternative strategies to conventional antibiotics, such as the use of non-tannin sorghum, may help maintain animal health due to its potential health-promoting properties. Our findings indicate that the AM activity of sorghum grain against *C. perfringens* is associated with a selective metabolic composition rather than the overall accumulation of defense-related metabolites. Additionally, considering sorghum as a feed source, the constitutive bioactive compounds may be more relevant than the inducible metabolites, as the grain used for poultry may not undergo significant pathogen-induced changes (*i.e.*, not all sorghum is challenged by fungal pathogens across various production environments).

The digestion process in the gut may affect metabolite bioactivity and bioavailability. In this study, a small subset of metabolites was consistently associated with AM activity against the bacterium, with higher concentrations of the hydroxycinnamic acid-derived C0540 and the lipid C0916 being the most significant ones. The identification of a major-effect QTL on chr01 associated with five metabolites with contrasting behavior and derived from different biosynthesis pathways further supports the idea that this region harbors key regulatory gene(s) that control diverse metabolic pathways that yield bioavailable AM compounds. These results provide a foundation for further investigation into the genetic and metabolic factors underlying AM activity in sorghum. While promising, additional validation is needed before developing genetic markers and biomarkers for sorghum breeding programs to increase AM activity in the grain.

## Data Availability

The datasets presented in this study can be found in online repositories. The names of the repository/repositories and accession number(s) can be found below: https://doi.org/10.6084/m9.figshare.31382908.
