# Supplementary material for: Untargeted metabolomics of mature sorghum [Sorghum bicolor (L.) Moench] grain reveals metabolites associated with antimicrobial activity against Clostridium perfringens
Source: Front Plant Sci. 2026 May 11;17:1821654. doi: 10.3389/fpls.2026.1821654 (PMC13199247; doi:10.3389/fpls.2026.1821654)
Supplement: Supplementary file 2 [file DataSheet2.pdf]

Supplementary Material

1. Data processing parameters.

The software XCMS (Smith 2006, Tautenhahn 2008) version 3.22.0 was used to process raw data using R v4.3.1. The following processing steps were used: (1) Peak detection (CentWave) : ppm = 25 peakwidth = c(2.2, 15), snthresh = 0, prefilter = c(5, 1000), mzCenterFun = wMean, integrate = 1, mzdiff = 0.01, fitgauss = TRUE, noise = 2, verboseColumns = TRUE, roiList = list(), firstBaselineCheck = TRUE, roiScales = numeric(0), extendLengthMSW = TRUE. (2) Retention time correction (Obiwarp) : binSize = 1 centerSample = integer(0), response = 10, distFun = cor_opt, gapInit = numeric(0), gapExtend = numeric(0), factorDiag = 2, factorGap = 1, localAlignment = FALSE, initPenalty = 0, subset = integer(0), subsetAdjust = average. (3) Peak grouping (PeakDensity) : bw = 1.75, minFraction = 0.3, minSamples = 1, binSize = 0.015, maxFeatures = 50. (4) Missing peak filling (FillChromPeaks) : expandMz = 0, expandRt = 0, ppm = 0, fixedMz = 0, fixedRt = 0.

Features were clustered using the *ramclustR* algorithm (Broeckling et al., 2014). Parameter settings were as follows: st = 5.03, sr = 0.5, maxt = 503, deepSplit = FALSE, hmax = 0.3, minModuleSize = 2, and cor.method = pearson.

Parameters for *do.findmain* were set to: mode = positive, mzabs.error = 0.005, ppm.error = 10, ads = [M+H]+ [M+Na]+ [M+K]+ [M+NH4]+ [2M+H]+ [2M+Na]+ [2M+K]+ [2M+NH4]+ [3M+H]+ [3M+Na]+ [3M+K]+ [3M+NH4]+, nls = [M+H-COCH2]+ [M+H-C2H3NO]+ [M+H-H2O]+ [M+H-NH3]+ [M+H-HCOOH]- [M+H-C6H12O6]+ [M+H-C5H10O5]+ [M+H-C12H22O11]+.

2. Supplementary Figures and Tables

2.1. Supplementary Figures

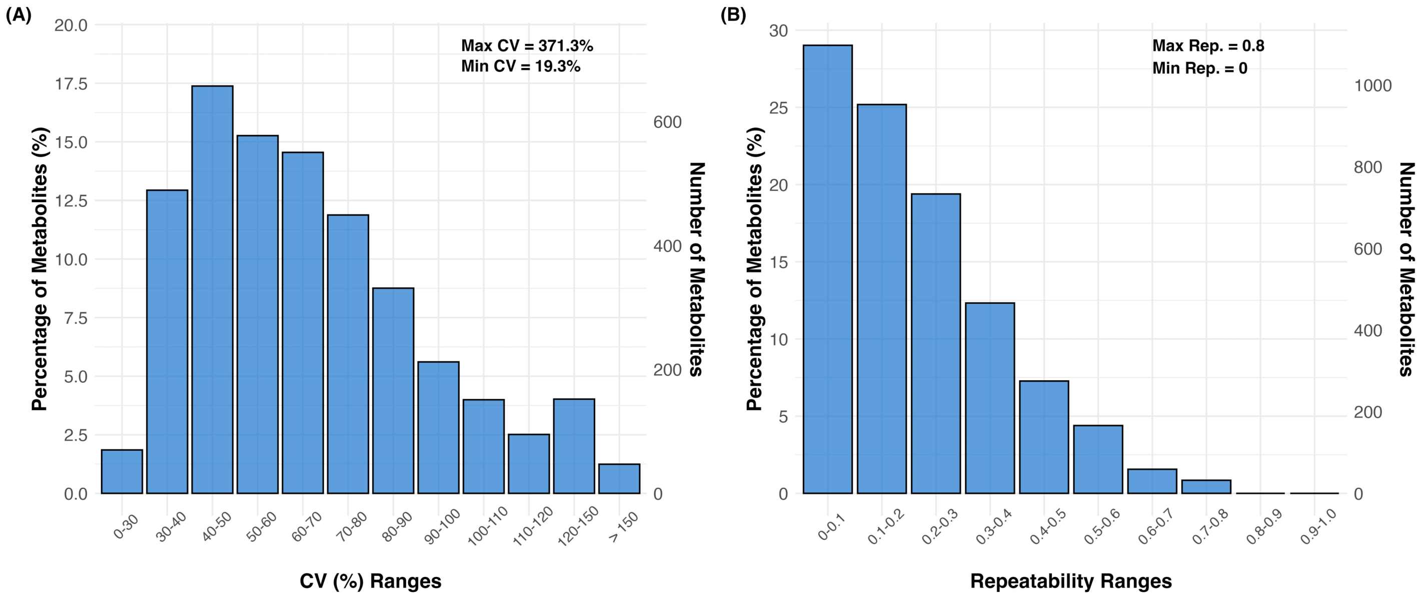

Supplementary Figure 1. Histograms showing the percentage (%) and number of metabolites within each bin range of (A) coefficients of variation (CV) and (B) repeatability for the 3,780 metabolites detected in the population.

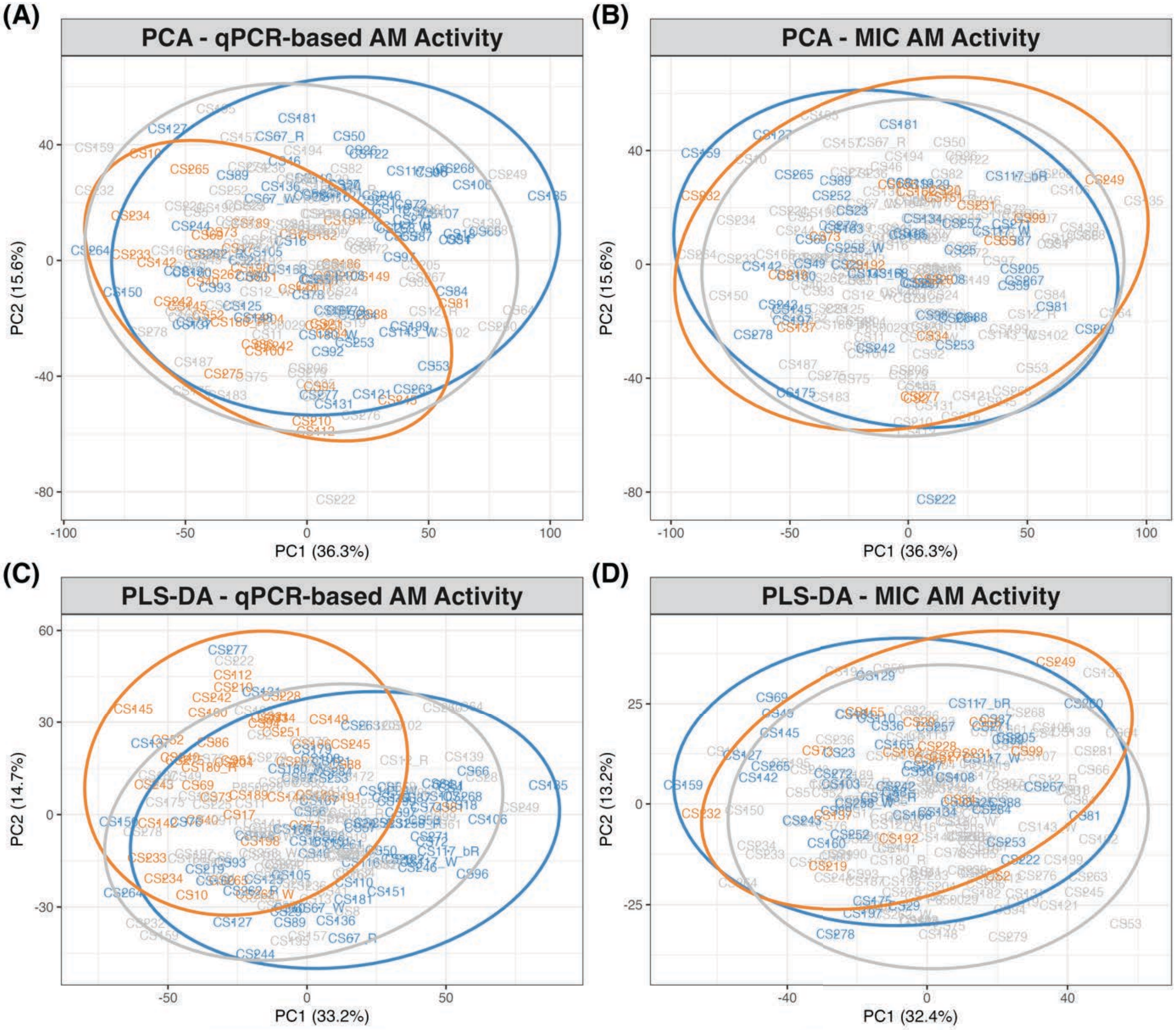

(C)

Metric	ER	pER
PC1	0.53	0.58
PC2	0.49	0.58

(D)

Metric	ER	pER
PC1	0.33	0.67
PC2	0.35	0.65

AM Activity ● High AM Activity ● Low AM Activity ● Medium AM Activity

Supplementary Figure 2. PCA (A-B) and PLS-DA (C-D) plots of all RILs, highlighting qPCR-based (A and C) and MIC (B and D) AM activity grouping (High AM, Medium AM and Low AM), showed no clear group separation. For the PLS-DA models, the tables show the error rate (ER) from a 10-fold cross-validation test and the ER for the permutation test (pER) for the first two PC. The Mfold method used was repeated 50 times, and the permutation test used 1,000 permutations.

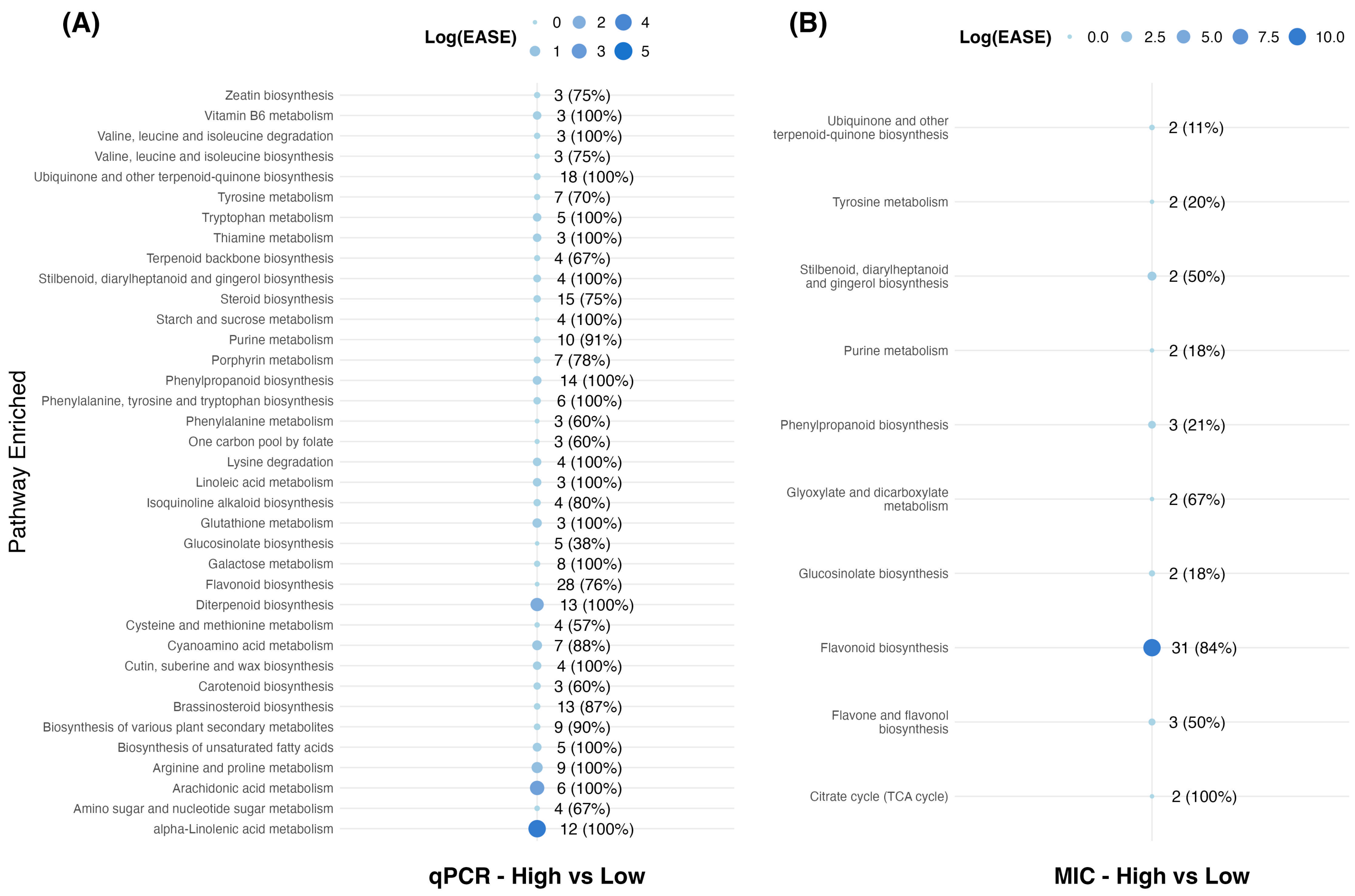

Supplementary Figure 3. Enriched pathways analyzing only High AM and Low AM activity groups for (A) qPCR and (B) MIC assays. The numbers next to each point indicate the significant compounds and the percentage of the total compounds that mapped to that pathway.

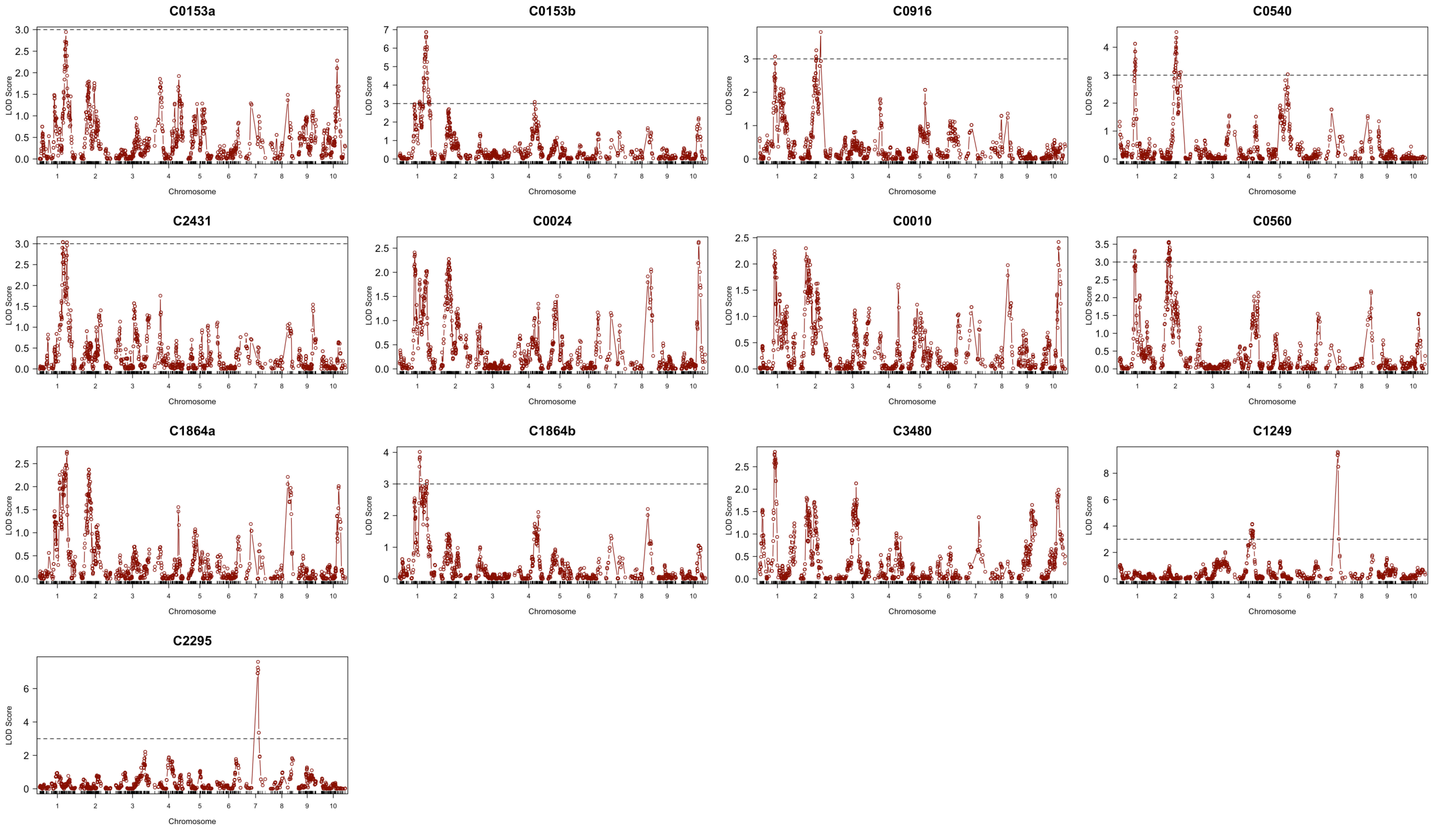

Supplementary Figure 4. Manhattan plots for the selected metabolites identified significant peaks for eight of the 13 compounds. The LOD scores (y-axis) are plotted against the position in the chromosome (x-axis). The horizontal dashed lines indicate the LOD score threshold for significance.

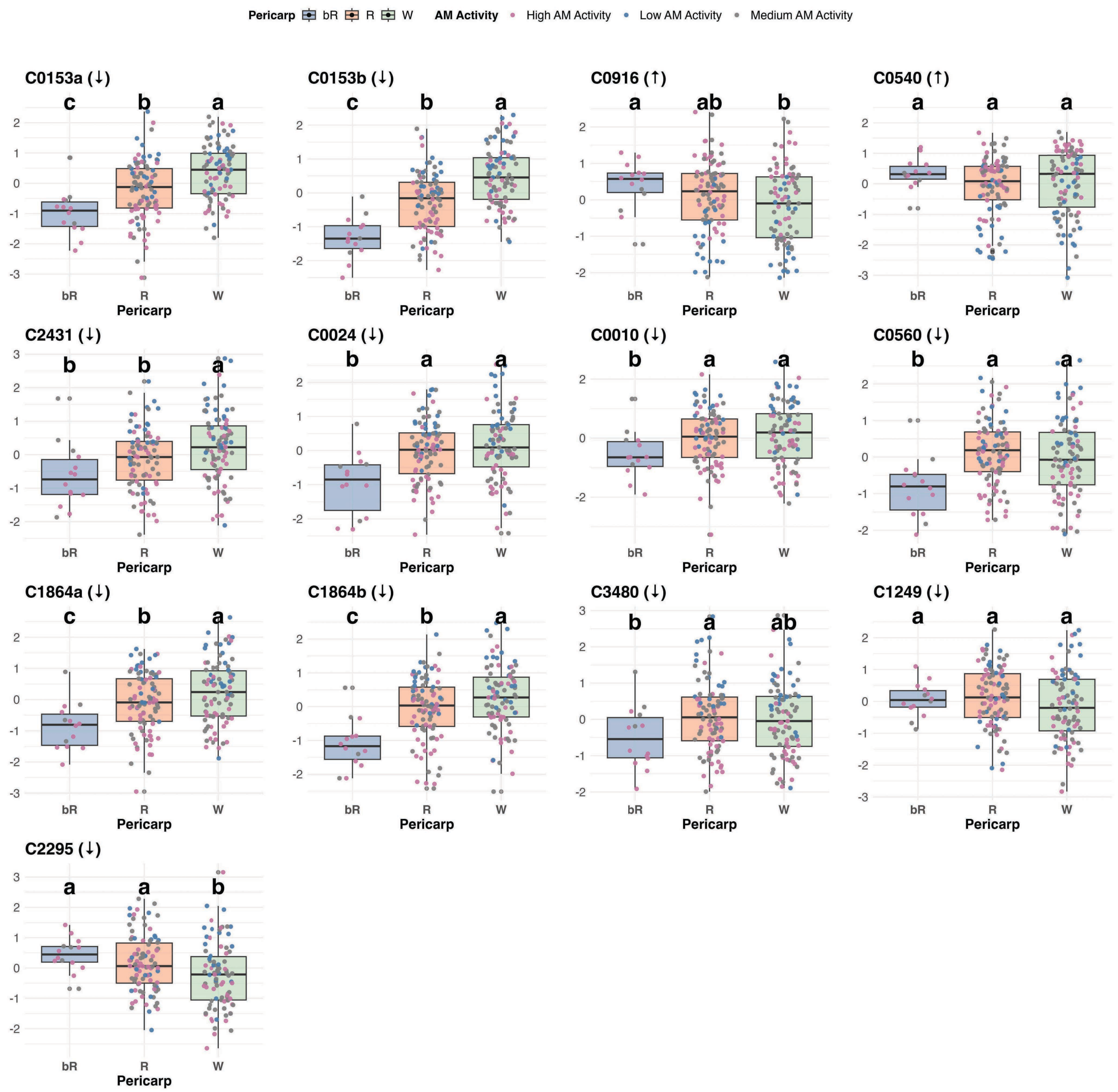

Supplementary Figure 5. Relative intensities by pericarp color of the 13 selected metabolites, indicating significant differences based on LSD test (*p*-value < 0.05). Each point represents the mean intensity value for a RIL, with the color indicating the AM activity group. The arrow next to each metabolite name indicates if its relative abundance was higher (↑) or lower (↓) in the High AM group.

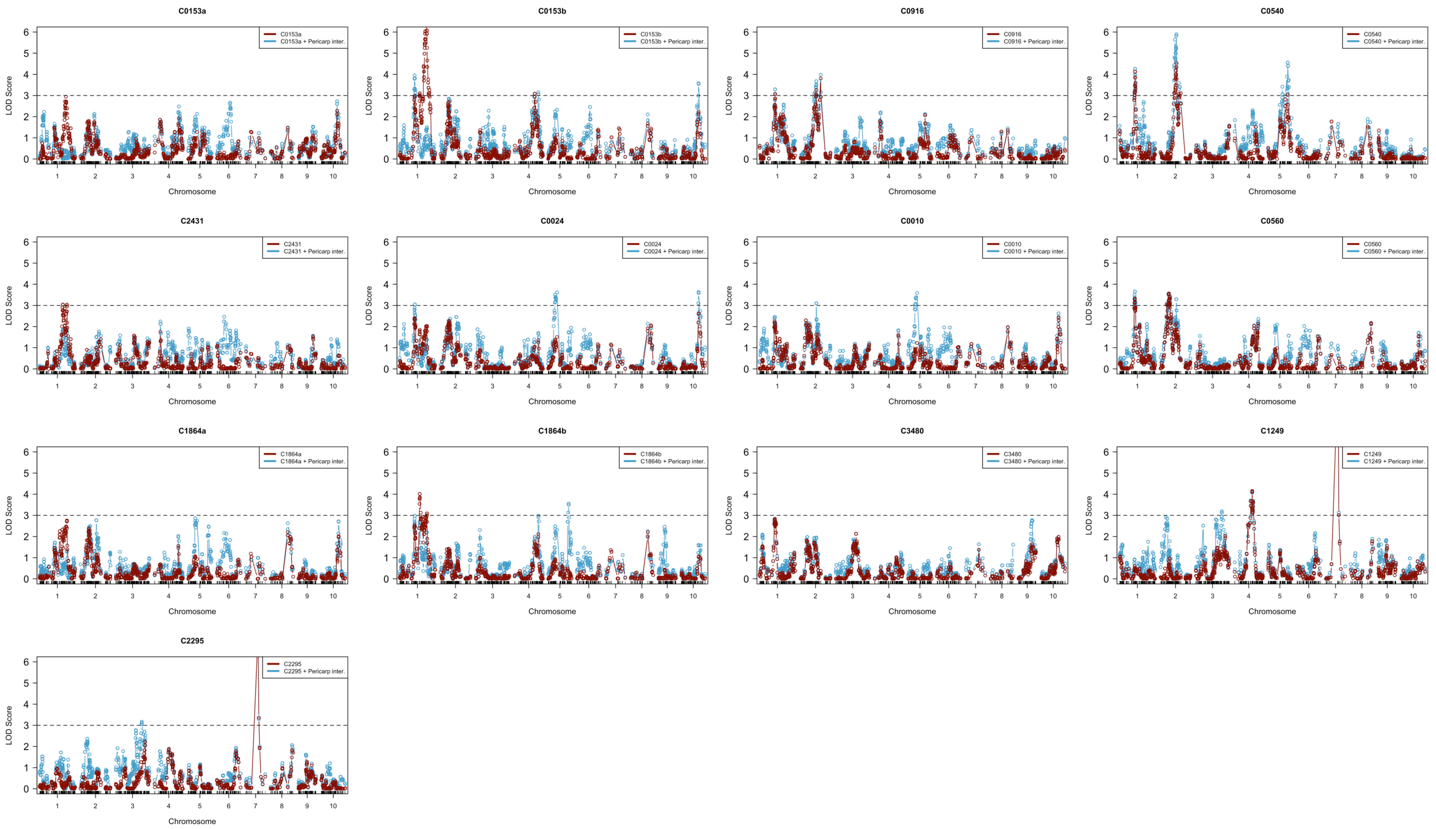

Supplementary Figure 6. Manhattan plots for the selected metabolites comparing models with and without pericarp color as an interactive covariate. The LOD scores (y-axis) are plotted against the position in the chromosome (x-axis). The horizontal dashed lines indicate the LOD score threshold for significance.

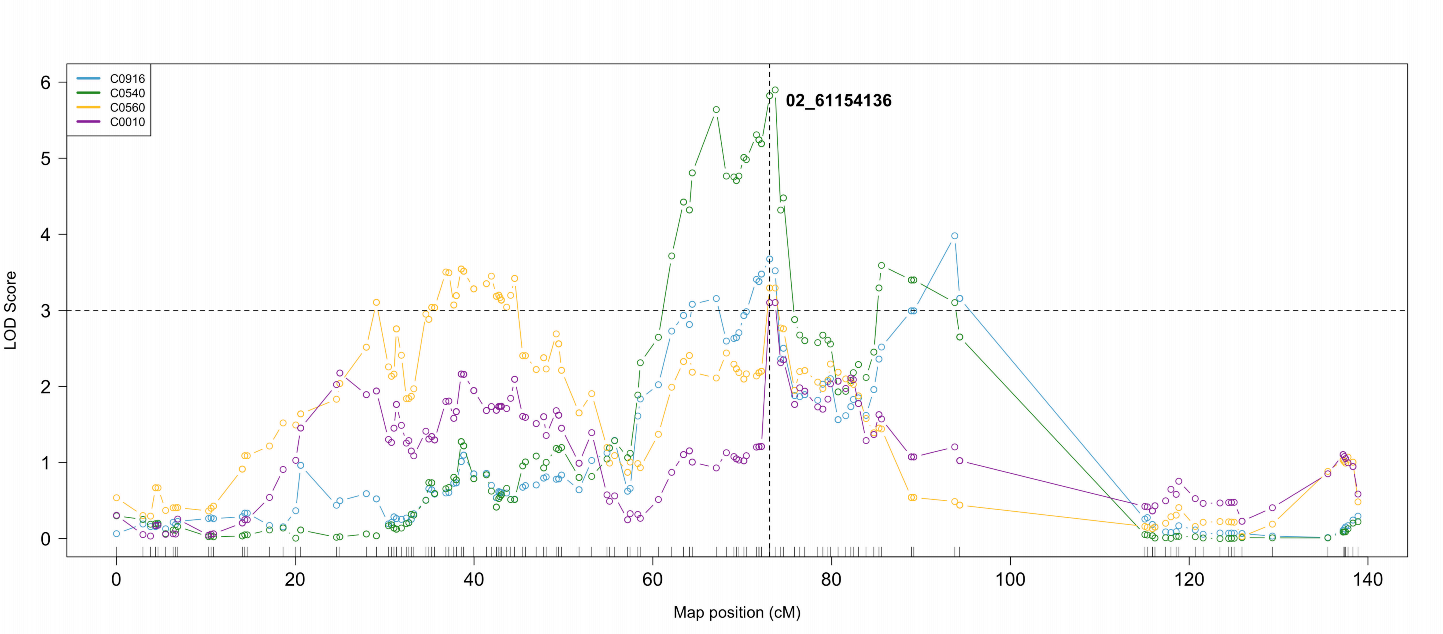

Supplementary Figure 7. Manhattan plot of chromosome 2 showing four metabolites. The vertical dashed line indicates the marker used for analysis. The LOD scores (y-axis) are plotted against the position in the chromosome (x-axis). The horizontal dashed line indicates the LOD score threshold for significance.

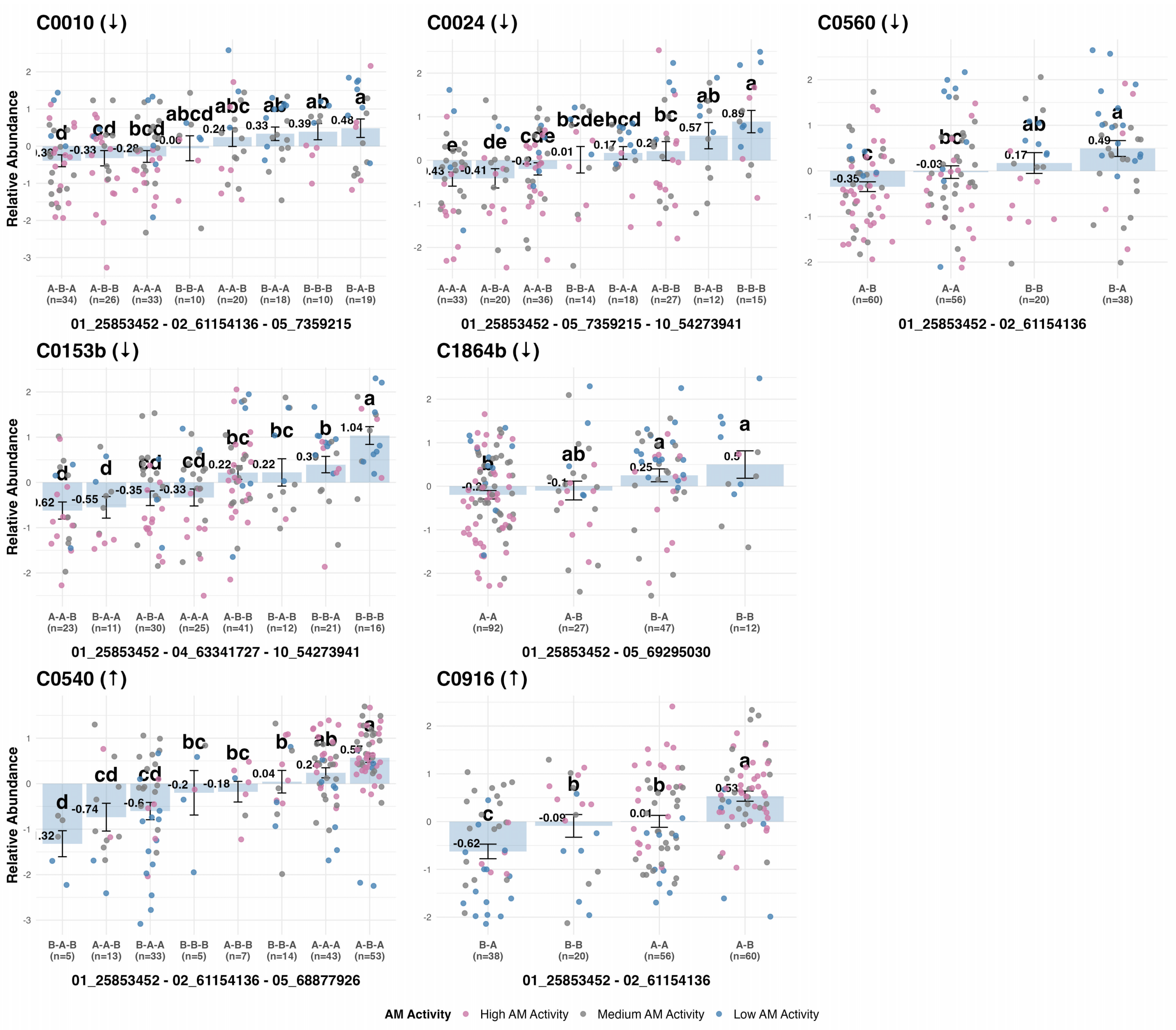

Supplementary Figure 8. Barplots of relative intensities by allele combination of three-loci and two-loci models for metabolites of interest. Each bar represents an allele combination reporting the number of RILs (n), mean values and statistical differences based on the LSD test (*p*-value < 0.05). Individual RILs are represented as points, colored by AM activity group. The A allele represents Tx2911, and the B allele represents P850029.

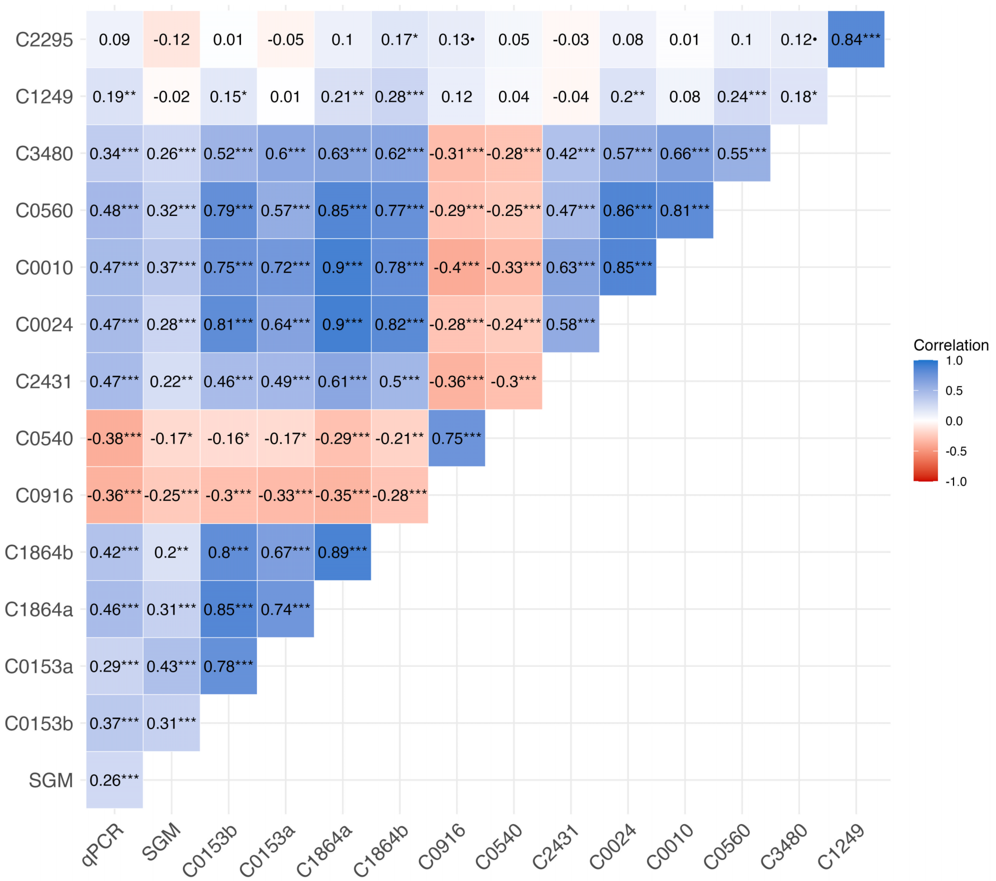

Supplementary Figure 9. Correlation matrix of the 13 selected metabolites, qPCR AM activity, and SGM pressure showed that these metabolites (except for C0153a) were strongly correlated with AM activity *against C. perfringens* rather than with grain mold (*i.e.*, a complex of fungi).

1.1. Supplementary Tables

Supplementary Table 1. Summary of the variance explained (%) by the first two principal components (PC1 and PC2) for different multivariate analyses across AM activity assays (qPCR and MIC) and pericarp color classification. The table shows that the first two PC explained approximately half of the variance observed in the metabolic profiles. For PLS-DA, only extreme categories were considered (*i.e.*, High AM vs. Low AM, and Pigmented vs. White).

		qPCR *		MIC *		Pericarp Color *
	PCA†	PLS-DA	PLS	PLS-DA	PLS	PLS-DA
**PC1**	36.3	33.6	31.4	26.0	31.6	25.3
**PC2**	15.6	15.2	16.8	18.0	7.3	19.4
**Total**	51.9	48.8	48.2	44.0	38.9	44.7

* Extremes (*i.e.*, High vs Low and Pigmented vs White).

† Full dataset (all RILs and all metabolites).

Supplementary Table 2. Summary of 10-fold cross-validation and permutation test for PLS-DA and PLS models showing the error rate (ER), ER for the permutation test (pER) the area under the curve (AUC), the AUC for the permutation test (pAUC), Q^2, R^2 and root-mean-squared error of prediction (RMSEP) values for the first two PC. The Mfold method used was repeated 50 times, and the permutation test used 1,000 permutations.

	PLS-DA											PLS					
	qPCR				MIC				Pericarp Color			qPCR			MIC		
	ER	pER	AUC	pAUC	ER	pER	AUC	pAUC	ER	AUC	pAUC	Q^2	R^2	RMSEP	Q^2	R^2	RMSEP
**PC1**	0.27	0.42	0.80	0.41	0.27	0.33	0.56	0.35	0.19	0.90	0.47	0.14	0.15	0.92	-0.15	0.04	1.07
**PC2**	0.22	0.43	0.85	0.48	0.27	0.37	0.60	0.50	0.03	0.996	0.53	0.03	0.06	0.89	-0.16	0.01	1.05

Supplementary Table 4. The 10-fold cross-validation for MIC AM activity optimal models sPLS-DA and sPLS showed that the metabolic signature is weak and inconsistent. For sPLS-DA, the number of variables, percentage of variance explained (%), ER and AUC for each PC are reported. For sPLS, the number of variables, percentage of variance explained (%), Q^2, R^2 and RMSEP for each PC are reported. The Mfold method used was repeated 50 times.

	sPLS-DA				sPLS				
	Variables	Variance	ER	AUC	Variables	Variance	Q^2	R^2	RMSEP
**PC1**	100	6.2	0.29	0.68	1	7.1	-0.05	0.004	1.02
**PC2**	1	6.1	0.25	0.68	1	6.1	-0.14	0.01	1.04

Supplementary Table 5. Summary of 10-fold cross-validation for qPCR AM activity optimal models sPLS-DA and sPLS. For sPLS-DA, the number of variables, variance explained by each PC (%), and AUC are reported. For sPLS, the number of variables, variance explained, Q2, R2 and RMSEP are reported. The Mfold method used was repeated 50 times.

	sPLS-DA				sPLS				
	**Variables**	**Variance**	**Error Rate**	**AUC**	**Variables**	**Variance**	**Q^2**	**R^2**	**RMSEP**
**PC1**	7	15.3	0.20	0.86	10	15.1	0.31	0.32	0.83
**PC2**	10	9.3	0.17	0.87	26	22.0	-0.10	0.01	0.81

Supplementary Table 6. Summary of the selected metabolites for AM activity based on sPLS-DA and sPLS, indicating the principal component for each analysis and compound stabilities extracted from the optimal model results. Stabilities <0.7 are highlighted in red.

**sPLS**	**PC1**	**Compound**	**C0153**	**C0916**	**C0540**	**C1585**	**C2431**	**C0024**	**C0010**	**C0560**	**C1864**	**C0289**
		**Stability**	1.000	0.998	0.982	0.976	0.908	0.880	0.784	0.732	0.704	0.664
**sPLS-DA**	**PC1**	**Compound**	**C0153**	**C0916**	**C3480**	**C1585**	**C0540**	**C0746**	**C1864**			
		**Stability**	1.000	0.998	0.894	0.734	0.724	0.524	0.328			
	**PC2**	**Compound**	**C1249**	**C2295**	**C3764**	**C2057**	**C2289**	**C0917**	**C2472**	**C1378**	**C2090**	**C1196**
		**Stability**	0.892	0.816	0.528	0.516	0.454	0.384	0.294	0.290	0.254	0.246

Supplementary Table 7. Summary table for the 12 selected metabolites (Met.) showing their *mz*, retention time (rt, s), VIP scores for PC1 of PLS-DA and PLS models, Log_2(FC) value (FC), relative abundance in the High AM group (Abun, high ↑ or low ↓), repeatability (Rep) and their annotations (if applicable).

Met.	*mz*	rt	PLS-DA	FC	PLS	Abun	Rep	Superclass	Class	Subclass	Direct parent
**C0010**	728.41	347.86	1.99	-1.03	2.20	↓	0.27	Organic acids and derivatives	Carboxylic acids and derivatives	Amino acids, peptides, and analogues	Cyclic peptides
**C0024**	1128.71	738.45	1.98	-1.31	2.24	↓	0.14				
**C0153**	407.09	329.48	2.21	-1.09	2.50	↓	0.29	Alkaloids and derivatives	Amaryllidaceae alkaloids	Lycorine-type amaryllidaceae alkaloids	Lycorine-type amaryllidaceae alkaloids
**C0540**	463.19	341.18	2.09	1.12	2.38	↑	0.42	Phenylpropanoids and polyketides	Flavonoids	Flavonoid glycosides	Flavonoid O-glycosides
**C0560**	365.08	398.10	1.90	-1.04	2.15	↓	0.23	Organoheterocyclic compounds	Benzofurans	Benzofuranones	Benzofuranones
**C0916**	447.19	361.30	2.28	1.64	2.38	↑	0.53	Phenylpropanoids and polyketides	Coumarins and derivatives	Unassigned	Coumarins and derivatives
**C1249**	345.04	393.56	0.83	-0.59	0.69	↓	0.48				
**C1585**	441.20	340.66	2.10	0.96	2.32	↑	0.29				
**C1864**	796.34	504.28	2.02	-1.07	2.20	↓	0.21				
**C2295**	375.09	303.42	0.79	-0.41	0.61	↓	0.40				
**C2431**	1173.77	738.04	1.96	-1.00	2.19	↓	0.20				
**C3480**	609.27	737.72	2.09	-1.34	2.18	↓	0.29				
**C0153a**	407.09	329.48		-0.89		↓	0.38				
**C0153b**				-0.97		↓	0.42				
**C1864a**	796.34	504.28		-1.10		↓	0.21				
**C1864b**				-0.96		↓	0.18				

Supplementary Table 8. Summary table for the 13 selected metabolites (Met.) showing their new annotations, the PubChem CID number and the molecular formula.

Met.	Superclass	Class	Subclass	Direct parent	PubChem ID	Formula	Annotation
**C0540 ↑**	Phenylpropanoids and polyketides	Cinnamic acids and derivatives	Hydroxycinnamic acids and derivatives	Hydroxycinnamic acids and derivatives	5321825	$C_{24}H_{28}N_2O_6$	Bis-ferulamidobutane
**C0916 ↑**	Lipids and lipid-like molecules	Glycerophospholipids	Glycerophosphates	1-acylglycerol-3-phosphates	52929751	$C_{19}H_{37}O_7P$	PA(16:1(9Z)/0:0)
**C0153b ↓**	Phenylpropanoids and polyketides	Macrolides and analogues	Unassigned	Macrolides and analogues	53320828	$C_{18}H_{21}ClO_7$	Pochonin N
**C0560 ↓**	Benzenoids	Benzene and substituted derivatives	Benzoic acids and derivatives	Hydroxybenzoic acid derivatives	6323491	$C_{18}H_{17}ClO_6$	Radicicol
**C0010 ↓**	Alkaloid				156012740	$C_{44}H_{55}N_3O_5$	CHEMBL4636434
**C0024 ↓**	Lipids and lipid-like molecules	Glycerophospholipids	Glycerophosphoglycero-phosphoglycerols	Cardiolipins	131823615	$C_{56}H_{104}O_{17}P_2$	CL(8:0/8:0/a-13:0/18:2(9Z,11Z))
**C0153a ↓**	Lipids and lipid-like molecules	Fatty Acyls	Fatty acyl glycosides	Fatty acyl glycosides of mono- and disaccharides	164215444	$C_{26}H_{49}NO_8$	HexCer 8:1;2O/12:0
**C1249 ↓**	Phenylpropanoids and polyketides	Coumarins and derivatives	Hydroxycoumarins	7-hydroxycoumarins	10925304	$C_{18}H_{10}O_6$	Edgeworin
**C1864a ↓**	Organoheterocyclic compounds	Naphthopyrans	Unassigned	Naphthopyrans	14166138	$C_{28}H_{39}NO_2$	2-(1,2,10-Trimethyl-6-oxa-23-azahexacyclo[12.10.0.02,11.05,10.016,24.017,22]tetracosa-16(24),17,19,21-tetraen-7-yl)propan-2-ol
**C2295 ↓**	Lignans, neolignans and related compounds	Unassigned	Unassigned	Lignans, neolignans and related compounds	146680	$C_{22}H_{14}O_6$	Elliptinone
**C2431 ↓**	Phenylpropanoids and polyketides	Macrolides and analogues	Unassigned	Macrolides and analogues	139583262	$C_{60}H_{105}N_3O_{18}$	N'-Methylniphimycin
**C3480 ↓**	Organoheterocyclic compounds	Tetrapyrroles and derivatives	Unassigned	Tetrapyrroles and derivatives	135474498	$C_{35}H_{36}N_4O_6$	10-Hydroxyphaeophorbide

Supplementary Table 9. Table showing the percentage of variance explained (PVE, %) by the peak marker on chromosome 1 for the 13 metabolites of interest, indicating the marker's name and position (cM).

Model: y ~ chr01 + Pericarp + chr01*Pericarp

Metabolite	Peak Marker	Pos. (cM)	PVE (%)
**C0024**	01_25095211	70.65087	10.5
**C0153b**	01_23064345	69.21405	22.8
**C0540**	01_25619342	71.52860	11.9
**C0560**	01_24808958	70.65085	9.3
**C0916**	01_25619342	71.52860	9.1
**C0010**	01_25095211*	70.65087	7.4
**C1864b**	01_25095211*	70.65087	12.2
**C0153a**	01_25095211*	70.65087	10.8
**C1864a**	01_25095211*	70.65087	8.7
**C3480**	01_25095211*	70.65087	7.3
**C2431**	01_25095211*	70.65087	5.1

* Marker used for analysis; no significant marker on chr01 (LOD < 3).
